# PACAP-PAC1R modulates fear extinction via the ventromedial hypothalamus

**DOI:** 10.1038/s41467-022-31442-w

**Published:** 2022-07-28

**Authors:** E. R. Velasco, A. Florido, Á Flores, E. Senabre, A. Gomez-Gomez, A. Torres, A. Roca, S. Norrholm, E. L. Newman, P. Das, R. A. Ross, A. Lori, O. J. Pozo, K. J. Ressler, L. L. Garcia-Esteve, T. Jovanovic, R. Andero

**Affiliations:** 1 Institut de Neurociències, Universitat Autònoma de Barcelona, Cerdanyola del Vallès, Barcelona Spain; 2 Departament de Psicobiologia i de Metodologia de les Ciències de la Salut, Universitat Autònoma de Barcelona, Cerdanyola del Vallès, Barcelona Spain; 3 Departament de Biologia Cel·lular, Fisiologia i Immunologia, Universitat Autònoma de Barcelona, Cerdanyola del Vallès, Barcelona Spain; 4 Laboratory of Neuropharmacology-NeuroPhar, Department of Experimental and Health Sciences, University Pompeu Fabra, Barcelona, Spain; 5 Integrative Pharmacology and Systems Neuroscience Research Group, Neurosciences Research Program, IMIM (Hospital del Mar Medical Research Institute), Barcelona, Spain; 6 Perinatal Mental health Unit, Department of Psychiatry and Clinical Psychology, Institute of Neuroscience, Hospital Clínic, IDIBAPS, Barcelona, Spain; 7 Programme for the Prevention and Treatment of Psychic Effects in Sexually Assaulted Women. Hospital Clínic de Barcelona, Barcelona, Spain; 8 Department of Psychiatry and Behavioral Neuroscience, Wayne State University, Detroit, MI USA; 9 McLean Hospital, Department of Psychiatry, Harvard Medical School, Belmont, MA USA; 10 Department of Neuroscience, Albert Einstein College of Medicine, Psychiatry Research Institute of Montefiore and Einstein, New York, NY USA; 11 Department of Psychiatry & Behavioral Sciences, Emory University, Atlanta, GA USA; 12 Centro de Investigación Biomédica En Red en Salud Mental (CIBERSAM), Instituto de Salud Carlos III, Madrid, Spain; 13 Unitat de Neurociència Traslacional, Parc Taulí Hospital Universitari, Institut d’Investigació i Innovació Parc Taulí (I3PT), Institut de Neurociències, Universitat Autònoma de Barcelona, Cerdanyola del Vallès, Spain; 14 ICREA, Barcelona, Spain; 15 Present Address: American Cancer Society, Inc., Atlanta, GA USA

**Keywords:** Fear conditioning, Neuroscience

## Abstract

Exposure to traumatic stress can lead to fear dysregulation, which has been associated with posttraumatic stress disorder (PTSD). Previous work showed that a polymorphism in the PACAP-PAC1R (pituitary adenylate cyclase-activating polypeptide) system is associated with PTSD risk in women, and PACAP (*ADCYAP1*)-PAC1R (*ADCYAP1R1*) are highly expressed in the hypothalamus. Here, we show that female mice subjected to acute stress immobilization (IMO) have fear extinction impairments related to *Adcyap1* and *Adcyap1r1* mRNA upregulation in the hypothalamus, PACAP-c-Fos downregulation in the Medial Amygdala (MeA), and PACAP-FosB/ΔFosB upregulation in the Ventromedial Hypothalamus dorsomedial part (VMHdm). DREADD-mediated inhibition of MeA neurons projecting to the VMHdm during IMO rescues both PACAP upregulation in VMHdm and the fear extinction impairment. We also found that women with the risk genotype of *ADCYAP1R1* rs2267735 polymorphism have impaired fear extinction.

## Abstract (web summary)

The PACAP-PAC1R neuropeptide system regulates neuroendocrine stress responses. Here the authors demonstrate a role for PACAP-PAC1R in ventromedial hypothalamus in modulating fear extinction.

## Introduction

Exposure to traumatic stress is one of the main environmental factors leading to disease^1^. Post-traumatic stress disorder (PTSD) is a debilitating mental disorder that occurs in some individuals exposed to traumatic stress. Moreover, PTSD may involve different symptoms including flashbacks, intrusive thoughts, and fear processing alterations^2,3^. PTSD prevalence ranges from 3.9 to 20%, and sociodemographic risk factors are key for its development^4,5^.

Importantly, women have a twofold likelihood of developing PTSD compared to men^6,7^. It is widely demonstrated that sex hormones in women are crucial for the maintenance and modulation of PTSD and its symptomatology^8–10^. However, only two prior studies have addressed the question of whether the specific phase of the menstrual cycle at the moment of trauma matters for the development of PTSD. Trauma experienced during the luteal phase appears to be associated with increased flashbacks but without increasing overall PTSD severity^11^. In contrast, no effect of the menstrual cycle phase during trauma was found in people with PTSD^12^. Nevertheless, both studies have some methodological limitations inherent to retrospective analyses and included women regardless of having irregular menstrual cycles or taking hormonal contraceptives.

The PACAP (encoded by *ADCYAP1*)-PAC1R (encoded by *ADCYAP1R1*) (pituitary adenylate cyclase-activating polypeptide and its type I receptor) neuropeptide system regulates neuroendocrine stress responses^13,14^. The rs2267735 single nucleotide polymorphism (SNP) in *ADCYAP1R1*, located within an estrogen response element, has been suggested as a specific biomarker of PTSD in women but not men^15^. In these women, the CC genotype in the rs2267735 is associated with lower *ADCYAP1R1* expression and higher PTSD symptoms, possibly resulting from differential SNP-dependent estrogen receptor transcriptional regulation of *ADCYAP1R1*^16^. Moreover, rs2267735 regulates amygdala and hippocampus response to threatening stimuli in women with PTSD^17^.

An important feature of PTSD is the impaired fear extinction (FE) to both stimuli related to trauma and newly aversive stimuli unrelated to trauma. Along this line, animal models are a valuable tool to uncover molecular mechanisms of FE and PTSD-like symptomatology. For example, we have previously shown that acute stress immobilization (IMO) impairs FE in male mice^18^ and shares molecular mechanisms with people with PTSD^19^. With this model, researchers have studied some of the mechanisms of traumatic stress, although it is important to note that such models are most likely insufficient to capture features of complex forms of trauma in humans, such as sexual assault. The inclusion in research studies of people who have experienced such trauma has the potential to help better understand the development of PTSD and inform about relevant variables to consider to support patients. Such studies must be planned in a context of an ethical and sensitive framework to avoid exacerbating the effects of past traumatic experiences.

Studies of PACAP-PAC1R system in female rodents have identified the bed nucleus of the stria terminalis (BNST) in mice^16^, and the prefrontal cortex in rats^20^, as important structures for fear processing. Notably, the highest levels in the brain of PACAP-PAC1R are in the hypothalamus^21,22^, but this brain region has not been associated with PTSD or fear processing.

Male lab animals are typically used in research studies focused on the neurobiological responses to stress. Female mice have often been disregarded with the argument of increased variability, given their cyclical hormonal fluctuations^23^. Despite this argument having been debunked, it is still held as a false construct in some cases^24–26^. Here, we used translational approaches to investigate the association of traumatic stress with hormonal cycle phases. Also, we explored the molecular mechanisms driving FE impairments in female mice subjected to IMO. We observed altered PACAP expression and neuronal activation in the ventromedial hypothalamus (VMHdm) and Medial Amygdala (MeA) that could be rescued by the inhibition of MeA to VMHdm projections during acute stress in female mice. In addition, to incorporate a translational connection to trauma in human participants, we investigated the association of a PAC1R genetic polymorphism with FE in a group of women that experienced trauma.

## Results

### IMO stress in female mice induces deficits in fear extinction regardless of the estrous cycle phase at trauma

**Fig. 1:**
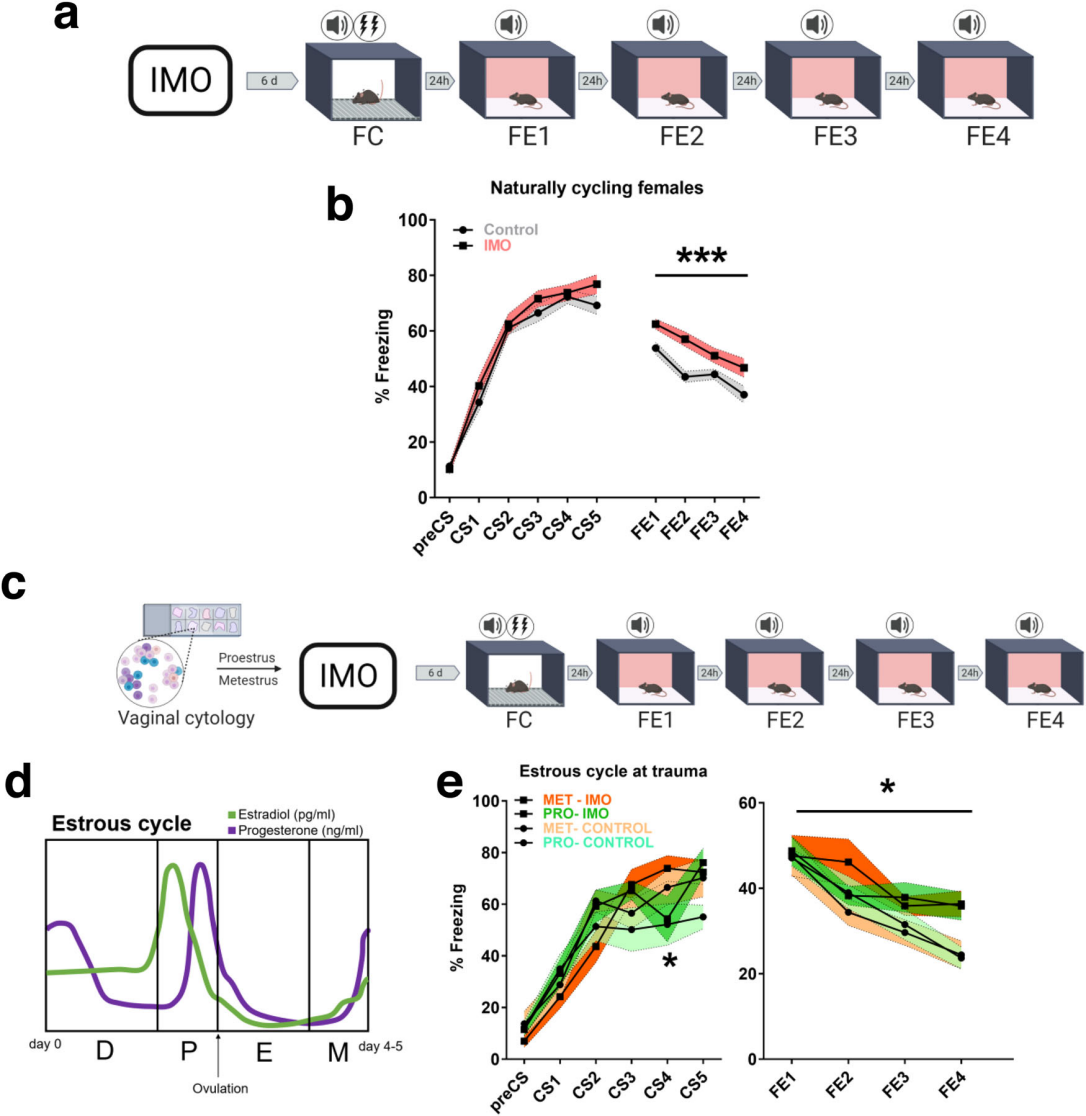
Long-lasting alterations of behavior after trauma and its association with the estrous cycle phase. **a** Schematic representation of the behavioral protocol. **b** Fear learning and fear extinction in naturally cycling females undergoing IMO or compensatory handling (control: *n* = 31, IMO: *n* = 32) (*P* = 0.001). **c** Methods used to monitor the estrous cycle before the behavioral procedures. **d** Estrous cycle and related hormonal levels in female C57BL/6 mice. **e** Fear learning, and fear extinction in proestrus or metestrus females (*n* = 7 per group) (*P* = 0.029). Data are expressed as mean ± SEM. *P ≤ 0.05, ***P ≤ 0.001. In **b**, **e**, main effect stress, main effect estrous cycle, main effect FE session or CS, and FE session* stress or CS*stress, estrous cycle*stress, FE session * estrous cycle, FE session * stress * estrous cycle interactions were analyzed using repeated-measures ANOVA, Bonferroni corrections were made for multiple comparisons. Asterisks above a line indicate significant main effect stress in repeated-measures ANOVA. CS conditioned stimulus, D diestrus, E estrus, FC fear conditioning, FE fear extinction session, IMO immobilization stress, M metestrus, Met metestrus, P proestrus, Pro proestrus. Source data are provided as a Source Data file.

We showed previously that IMO produces impairments in FE in male mice, a deficit that is also observed in people with PTSD^19^. Here, we explored whether IMO exposure induced impairments in FE in naturally cycling female mice by exposing them to IMO or compensatory handling (control group) for 5 min and subjecting them 6 days later to a fear conditioning (FC) task followed by four consecutive FE tests (Fig. 1a). Results showed that female mice exposed to IMO had greater freezing rates during the FE tests compared to the control, non-stressed group (*F*(1,61) = 12.528, *P* = 0.001) (Fig. 1b). Results in male mice were on the same line (Supplementary Fig. 1a, b). The effect of IMO was specific for FE tests in both sexes since no significant interactions with stress were observed during FC.

Changes in bodyweight in rats after IMO are considered a marker of stressor intensity^27,28^. In our experiments, IMO exposure resulted in decreased weight gain in males and females, with female mice gaining overall less weight than males (*χ*^2^(3) = 36.484, *P* < 0.001, control males vs IMO males, *P* = 0.020; control females vs IMO females, *P* < 0.001, control females vs control males, *P* = 0.007, IMO females vs IMO males, *P* = 0.006) (Supplementary Fig. 1c). Repeated-measures analyses showed that males had their weight gain decreased by IMO (*F*(1,20) = 16.952, *P* = 0.001; control t1 vs IMO t1, *P* = 0.09; control t2 vs IMO t2, *P* = 0.009; control t1 vs control t2, *P* < 0.001; IMO t1 vs IMO t2, *P* = 0.009) (Supplementary Fig. 1d), but female mice experienced a slight weight loss (*F*(1,37) = 8.937, *P* = 0.005; control t1 vs IMO t1, *P* = 0.065; control t2 vs IMO t2, *P* = 0.690; control t1 vs control t2, *P* = 0.038; IMO t1 vs IMO t2, *P* = 0.045) (Supplementary Fig. 1e). Moreover, some evidence suggests that stress can increase grooming^29^, but we found no differences in the number of grooming episodes in IMO or control female mice during FE1 (Supplementary Fig. 1f).

Female hormonal cycles modulate neurotransmitter and hypothalamic–pituitary–adrenal axis (HPA) function and affect spatial learning, short-term memory tasks, and FE consolidation^30–32^. Still, the interaction of stress with the hormonal cycles is less clear, especially to answer the question of whether the estrous cycle phase at the moment of trauma is associated with a greater vulnerability to develop a more severe post-traumatic behavioral phenotype. We monitored the estrous cycle of a group of naturally cycling female mice with vaginal smear cytology and subjected them to IMO during the proestrus (high estradiol/high progesterone) and metestrus (low hormones) phases of the estrous cycles (Fig. 1c, d). These phases were selected based on previous research^33^. We used the estrous cycle and stress as between-subject variables and FE sessions as within-subject variables. We found a main effect of stress (*F*(1,24) = 5.419, *P* = 0.029) without a main effect of cycle (*F*(1,24) < 0.001, *P* = 0.995), or an interaction cycle * stress (*F*(1,24) = 0.245, *P* = 0.625). Although, our analyses revealed slightly different freezing rates during FC given by an interaction of CS * cycle, specifically during CS4 (*F*(4,96) = 2.644, *P* = 0.038; CS4 proestrus vs metestrus *P* = 0.042) and independently from stress (*F*(4,96) = 1.197, *P* = 0.317) (Fig. 1e and Supplementary Fig. 1g). This indicates that the estrous cycle phase at the time of trauma is not associated with differences in the increased freezing levels during FE induced by IMO. In addition, no changes in weight gain were observed in cycle monitored female mice between IMO and control groups (*t*(13) = 0.079, *P* = 0.938) (Supplementary Fig. 1h), neither in repeated measures analyses (*F*(1,13) < 0.001, *P* = 0.994) (Supplementary Fig. 1i).

Sex hormones have cyclical fluctuations that interact with stress hormone responses^31,34^. In rats, there are basal and stress-induced corticosterone concentrations that are higher in proestrus compared to estrus and diestrus^35^. Still, these differences seem to be constrained to an early corticosterone response to brief acute stressors^36^. Based on our findings regarding the estrous cycle, we hypothesized that differences in basal hypothalamic–pituitary-gonadal axis (HPG) hormonal levels would result in similar recovery profiles of HPA hormones. A group of female mice in proestrus or metestrus were subjected to IMO and hormonal regulation was assessed shortly after trauma (60 min) (Supplementary Fig. 2a). Regarding the HPA axis, corticosterone was robustly upregulated in IMO females regardless of the estrous cycle phase (*χ*^2^(3) = 116.159, *P* < 0.001; proestrus basal vs proestrus IMO, *P* < 0.001; metestrus basal vs metestrus IMO, *P* < 0.001; proestrus IMO vs metestrus IMO, *P* = 0.886) (Supplementary Fig. 2b). Additional steroids in the HPA axis, deoxycorticosterone (*χ*^2^(3) = 62.322, *P* < 0.001; proestrus basal vs proestrus IMO, *P* < 0.001; metestrus basal vs metestrus IMO, *P* < 0.001; proestrus IMO vs metestrus IMO, *P* = 0.570) (Supplementary Fig. 2c) and dehydrocorticosterone (*χ*^2^(3) = 139.334, *P* < 0.001; proestrus basal vs proestrus IMO, *P* < 0.001; metestrus basal vs metestrus IMO, *P* < 0.001; proestrus IMO vs metestrus IMO, *P* = 1.000) followed the same direction (Supplementary Fig. 2d).

Regarding the HPG axis, we found basal differences in progesterone levels between proestrus and metestrus (*χ*^2^(3) = 65.065, *P* < 0.001; proestrus basal vs metestrus basal, *P* < 0.001). After stress, the levels of progesterone decreased in the metestrus IMO group compared to metestrus basal (*P* < 0.001) with no changes in progesterone regulation after stress in proestrus (*P* = 1.000) (Supplementary Fig. 2e). There were differences in the basal testosterone levels (*χ*^2^(3) = 13.756, *P* = 0.003; proestrus basal vs metestrus basal, *P* = 0.046) and stress increased testosterone levels in metestrus females (metestrus basal vs metestrus IMO, *P* = 0.002), but not in proestrus females (proestrus basal vs proestrus IMO, *P* = 1.000) (Supplementary Fig. 2f). Furthermore, we found no differences in basal estradiol levels (*χ*^2^(3) = 9.410, *P* = 0.024; proestrus basal vs metestrus basal *P* = 0.280), but IMO decreased estradiol in proestrus females (proestrus basal vs proestrus IMO, *P* = 0.029), but not in metestrus females (metestrus basal vs metestrus IMO, *P* = 1.000) (Supplementary Fig. 2g). These results provide evidence for a robust upregulation of the HPA axis in IMO females in both estrous phases with similar 60 min post-stress hormonal profiles. Moreover, basal differences in HPG hormones showed a differential regulation by IMO.

### *Adcyap1- Adcyap1r1* upregulation is related to fear extinction deficits in a female PTSD-like mouse model

**Fig. 2:**
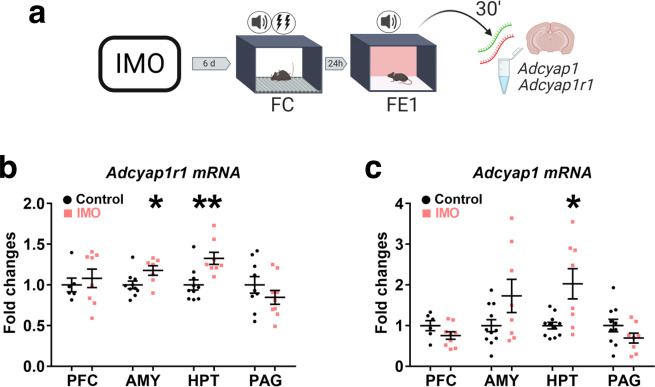
*Adcyap1*-*Adcyap1r1* regulation during early FE (FE1). **a** Representation of the methods used to evaluate *Adcyap1, Adcyap1r1* regulation after FE1. **b** *Adcyap1r1* mRNA regulation 30 min after FE1 in prefrontal cortex (PFC) (control: *n* = 6, IMO: *n* = 8), amygdala (AMY) (control: *n* = 10, IMO: *n* = 7) (*P* = 0.030), hypothalamus (HPT) (control: *n* = 10, IMO: *n* = 8) (*P* = 0.002), and periaqueductal gray (PAG) (control: *n* = 9, IMO: *n* = 9). **c** *Adcyap1* in PFC (control: *n* = 6, IMO: *n* = 9), AMY (control: *n* = 11, IMO: *n* = 8), HPT (control: *n* = 11, IMO: *n* = 8) (*P* = 0.020), and PAG (control: *n* = 10, IMO: *n* = 8). Data are means ± SEM. *P ≤ 0.05, **P ≤ 0.01. Two-tailed *t* tests were conducted. IMO immobilization stress, FC fear conditioning, FE fear extinction. Source data are provided as a Source Data file.

High PACAP levels and *ADCYAP1R1* genetic variants are related to greater post-traumatic symptoms and fear responses in women^15^. Further, *Adcyap1r1* mRNA is upregulated 2 h after FC in male mice^15,37^, but few studies have studied its regulation in female mice^38^. We exposed a group of naturally cycling female mice to IMO or compensatory handling for 5 min, 6 days later they took a FC task followed by one session of FE (FE1). After FE1 (30 min), brains were removed to study *Adcyap1-Adcyap1r1* mRNA regulation (Fig. 2a). We found greater freezing levels in female mice exposed to IMO (*F*(1,19) = 4.741, *P* = 0.042) (Supplementary Fig. 3a, b) and *Adcyap1r1* transcripts were upregulated in the amygdala (*t*(15)=−2.396, *P* = 0.030) and hypothalamus (*t*(16) = −3.586, *P* = 0.002) of IMO female mice (Fig. 2b). Similarly, *Adcyap1* mRNA was upregulated in the hypothalamus of IMO female mice (*t*(9.217) = −2.795, *P* = 0.020) (Fig. 2c). Moreover, mean freezing levels during FE1 were not correlated with *Adcyap1* or *Adcyap1r1* mRNA regulation in the amygdala (*Adcyap1r1*, *r* = −0.213, *P* = 0.411; *Adcyap1*, *r* = −0.075, *P* = 0.759) or hypothalamus (*Adcyap1r1*, *r* = −0.237, *P* = 0.343; *Adcyap1*, *r* = −0.240, *P* = 0.322) (Supplementary Fig. 3c). These results demonstrate that IMO exposure increased the regulation of *Adcyap1r1* transcripts in the hypothalamus–amygdala and *Adcyap1* in the hypothalamus of females after a FE test.

### Activity profiles of PACAP + neurons in the amygdala and hypothalamus are related to FE deficits observed after IMO

**Fig. 3:**
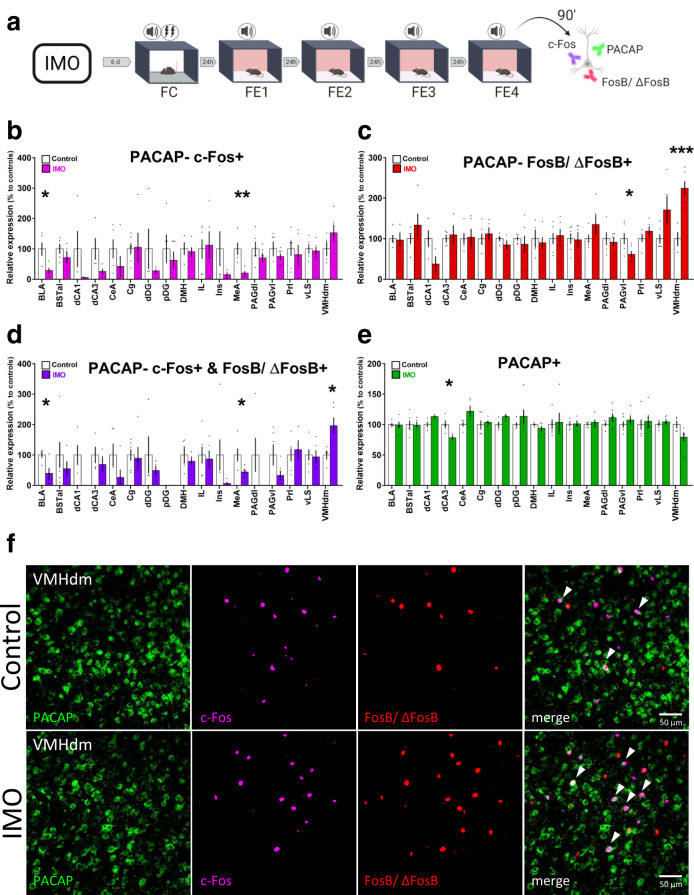
Immobilization stress induces changes in PACAP+ neuronal activation after a fear extinction task. **a** Schematic representation of the behavioral and immunohistochemical methods. **b** Quantification of cellular colocalization of PACAP-c-Fos+ (BLA and MeA *n* = 5 per group) (BLA: *P* = 0.023, MeA: *P* = 0.005). **c** Quantification of cellular colocalization of PACAP-FosB/ΔFosB+ (PAGvl *n* = 6 per group, VMHdm control: *n* = 4, IMO: *n* = 6) (PAGvl: *P* = 0.037, VMHdm: *P* = 0.001). **d** Quantification of double colocalization of PACAP-c-Fos+ and FosB/ΔFosB+ (BLA and MeA *n* = 5 per group; VMH control: *n* = 4, IMO: *n* = 6) (BLA: *P* = 0.015, MeA: *P* = 0.040, VMHdm: *P* = 0.030). **e** Quantification of PACAP+ cells (dCA3 *n* = 4 per group) (dCA3: *P* = 0.028). **f** Representative confocal images of PACAP, c-Fos, FosB/ΔFosB colocalization in the VMHdm. White arrowheads signal triple colocalization. Scale bar 50 μm. Results are presented as relative expression compared to controls (in %) and extracted from cells per mm^2^ normalized to DAPI. Data are presented as means ± SEM. *P ≤ 0.05, **P ≤ 0.01, ***P ≤ 0.001. Two-tailed *t* tests or Mann–Whitney’s *U* tests were used. BLA basolateral amygdala, BSTal bed nucleus of the stria terminalis anterolateral part, dCA1 dorsal CA1, dCA3 dorsal CA3, CeA central amygdala, Cg cingulate cortex, dDG dorsal dentate gyrus, pDg posterior dentate gyrus, DMH dorsomedial hypothalamus, FC fear conditioning, FE fear extinction, IL infralimbic cortex, IMO immobilization stress, Ins insular cortex, MeA medial amygdala, PAGdl dorsolateral periaqueductal gray, PAGvl ventrolateral periaqueductal gray, Prl prelimbic cortex, vLS ventral lateral septum, VMHdm ventromedial hypothalamus dorsomedial nucleus. Source data are provided as a Source Data file.

Then, we subjected a group of naturally cycling female mice to the same behavioral task and collected tissue 90 min after FE4 to perform an immunofluorescence study (Fig. 3a). We used c-Fos+ and FosB/ΔFosB+ as markers of recent and repeated neuronal activity^39,40^, and colocalized them with PACAP + neurons. We first validated a PACAP antibody with a conditional KO approach and then quantified signal colocalization in areas implicated in IMO or FE according to previous research^41,42^ (Supplementary Fig. 4a–c). Results showed that shortly after the last FE session, IMO females had fewer PACAP-c-Fos+ neurons in the basolateral amygdala (BLA) (*t*(5.339) = 3.159, *P* = 0.023) and in the MeA (*t*(8) = 3.797, *P* = 0.005) compared to controls (Fig. 3b). In turn, we observed decreases in PACAP-FosB/ΔFosB+ in the ventrolateral periaqueductal gray (PAGvl) (*t*(10) = 2.397, *P* = 0.037) and a marked increase in the VMHdm (*t*(8) = −4.982, *P* = 0.001) (Fig. 3c). PACAP neurons with double colocalization, PACAP-c-Fos+ & FosB/ΔFosB+, mirrored the previous results with lower colocalization observed in the BLA (*t*(8) = 3.078, *P* = 0.015) and MeA (*t*(8) = 2.446, *P* = 0.040), and greater colocalization in the VMHdm (*t*(8) = −2.639, *P* = 0.030) (Fig. 3d). See Fig. 3f for a representative image. We also detected a reduction of PACAP+ signal in the dorsal CA3 of IMO females (*t*(6) = 2.893, *P* = 0.028) (Fig. 3e). Mean freezing levels during FE were negatively correlated with PACAP-c-Fos+ neurons in MeA (*r* = −0.716, *P* = 0.020) and positively correlated with PACAP-FosB/ΔFosB+ neurons in VMHdm (*r* = 0.715, *P* = 0.020) (Supplementary Fig. 4d–g). These results show that increased freezing levels during four FE sessions in IMO female mice are associated with decreased activation of PACAP+ neurons in MeA and repeated activation of PACAP+ neurons in the VMHdm.

### Inhibition of the MeA to VMHdm projections rescues fear extinction deficits and PACAP upregulation after IMO

**Fig. 4:**
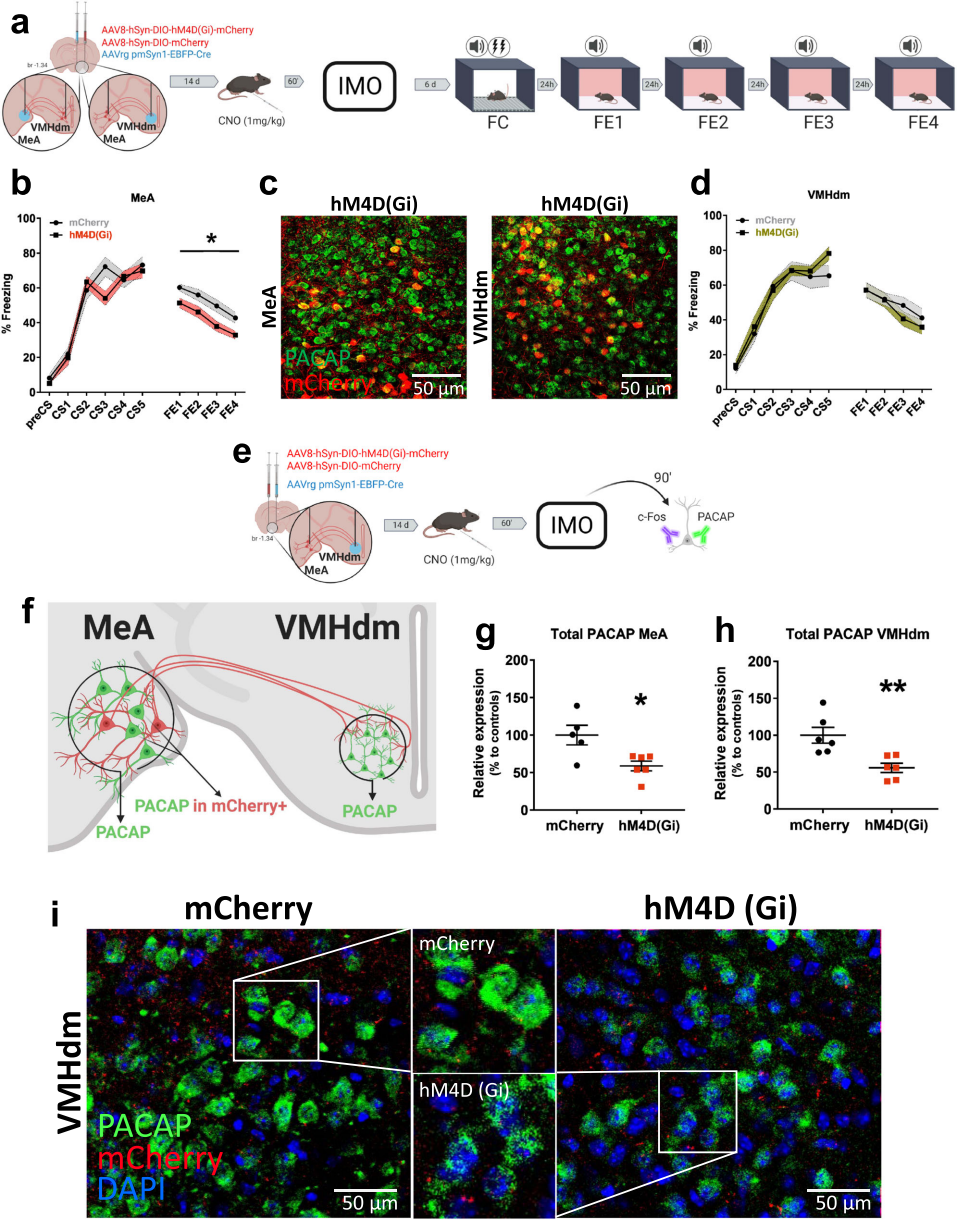
Effect of the chemogenetic inhibition of the medial amygdala-ventromedial hypothalamus circuit during immobilization stress. **a** Schematic representation of the methods and timeline of experiments. **b** Fear learning and fear extinction in mice with a temporal inhibition of MeA to VMHdm projections (hM4D(Gi)) during IMO vs controls (mCherry) (mCherry: *n* = 10, hM4D(Gi): *n* = 8) (*P* = 0.036). **c**, left panel: representative confocal images showing PACAP+ (green) neurons and mCherry+ (red) cell bodies in the MeA in animals receiving hM4D(Gi) in MeA; right panel: cell bodies in the VMHdm in animals receiving hM4D(Gi) in VMHdm. Scale bar 50 μm. **d** Fear learning, and fear extinction in mice with a temporal inhibition of VMHdm to MeA projections (hM4D(Gi)) during IMO vs controls (mCherry) (mCherry: *n* = 6, hM4D(Gi): *n* = 8). **e**, **f** Methods used to assess PACAP expression shortly after IMO in animals with inhibited MeA to VMHdm circuitry (hM4D(Gi) vs controls (mCherry)). **g**, **h** Effect of a temporal inhibition of MeA to VMHdm projections on PACAP expression shortly after IMO in the MeA (mCherry: *n* = 5, hM4D(Gi): *n* = 6) (*P* = 0.015) and VMHdm (mCherry: *n* = 6, hM4D(Gi): *n* = 6) (*P* = 0.005). Results are presented as relative expression compared to controls (in %) and extracted from PACAP levels normalized to DAPI (*n* = 5–6 per group). **i** Representative confocal images displaying differences in the expression of PACAP in the VMHdm of animals receiving a temporal inhibition of MeA to VMHdm projections during IMO (hM4D(Gi)) left panel vs controls (mCherry) right panel. Respective magnifications are shown in the middle part upper panel (mCherry) and lower panel (hM4D(Gi)). Scale bar: 50 μm. Data are means ± SEM. *P ≤ 0.05, **P ≤ 0.01. In **b**, **d**, main effect treatment, main effect FE session, and FE session * treatment interaction were analyzed using a repeated-measures ANOVA. Asterisks above a line indicate significant main effect treatment in repeated-measures ANOVA. In **g**, **h**, two-tailed *t* tests were used. CNO clozapine N-oxide, FC fear conditioning, FE fear extinction, IMO immobilization stress, MeA medial amygdala, VMHdm ventromedial hypothalamus dorsomedial nucleus. Source data are provided as a Source Data file.

Connections between the amygdala and the hypothalamus are involved in stress processing and fear learning. The MeA processes sensory cues and it is strongly activated by emotional stressors^43,44^. The VMHdm receives extensive projections from the MeA, and its repeated activity is related to persistent defensive behaviors in response to heterogeneous threats^45^. A MeA-VMH circuit was previously implicated in aggression and conspecific/predator threat responses, but not in emotional stressors^46,47^. Therefore, since it is known that IMO exposure strongly elicits an upregulation of c-Fos in the MeA^41,43^, we hypothesized that trauma would increase MeA activity and enhance signaling to the VMHdm to trigger increases in PACAP function, which would further promote VMHdm activation. However, since MeA-VMH share reciprocal connections and the amygdala is a possible inhibitory feedback mechanism for VMH function^48–50^, we tested for circuit directionality in separate groups of naturally cycling female mice. We used a non-PACAP-selective combined chemogenetic retrograde AAV approach to inhibit the activity of this circuit by injecting AAV-hM4Di-DREADD (AAV8-hSyn-DIO-hM4D(Gi)-mCherry) or AAV-DIO-mCherry (AAV8-hSyn-DIO-mCherry) and AAV5rg-pmSyn-EBFP-Cre, so that hM4D(Gi) receptors were expressed in VMHdm-projecting MeA neurons or MeA-projecting VMHdm neurons (Fig. 4a, c). mCherry and enhanced blue fluorescent protein (EBFP) reporters were used to verify injection sites (Supplementary Fig. 5). To trigger circuit inhibition, we administered Clozapine N-oxide (CNO, 1 mg/kg I.P.) 1 h before the exposure to IMO; 6 days later mice were exposed to a FC task followed by four consecutive FE tests (Fig. 4a). Our results showed that the deficits in FE induced by IMO were rescued by the inhibition of MeA to VMHdm projections during IMO (*F*(1,16) = 5.250, *P* = 0.036) (Fig. 4b), but not by the inhibition of VMHdm to MeA projections (*F*(1,12) = 0.196, *P* = 0.666) (Fig. 4d).

We used the same approach to study the short-term dynamics of PACAP after trauma in mice (90 min after IMO) (Fig. 4e, f). The overall PACAP levels were significantly lower in the MeA (*t*(9) = −2.99, *P* = 0.015) and VMHdm (*t*(10) = −3.544, *P* = 0.005) of animals that had MeA to VMHdm projections inhibited during IMO (Fig. 4g–i). However, PACAP levels were not significantly lower in mCherry+ neurons in the MeA (*t*(9) = −2.203, *P* = 0.055) (Supplementary Fig. 6a, b). Furthermore, inhibition of MeA to VMHdm projections resulted in a greater number of overall c-Fos+ (*U* = 1.500, *P* = 0.009) and PACAP-c-Fos+ neurons (*U* = 1.000, *P* = 0.009) in the MeA but not in mCherry+ MeA neurons (*U* = 15.500, *P* = 0.548) (Supplementary Fig. 6c, d, f). The chemogenetic inhibition did not change c-Fos+ (*U* = 17.500, *P* = 0.937) or PACAP-c-Fos+ (*t*(9) = −1.608, *P* = 0.142) expression in the VMHdm (Supplementary Fig. 6e, g).

**Fig. 5:**
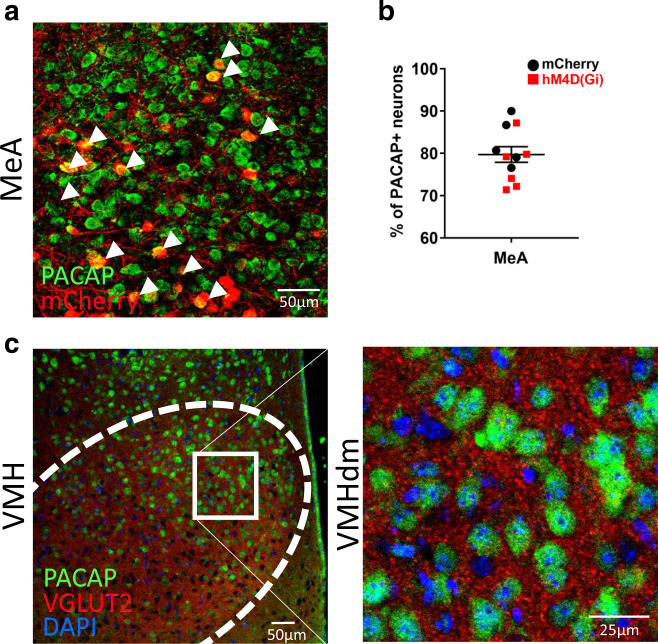
Sites of delivery of viral vectors in MeA and VMHdm. **a** Representative image showing the colocalization of mCherry+ neurons with PACAP+ immunolabeling in the MeA. White arrowheads signal colocalization. **b** Quantification of the proportion of mCherry+ cells with PACAP+ immunolabeling of animals receiving AAV5rg-pmSyn-EBFP-Cre in the VMHdm and either AAV-hM4Di-DREADD or AAV-DIO-mCherry in the MeA (mCherry: *n* = 5, hM4D(Gi): *n* = 6). **c** Representative image showing a prominent VGLUT2 expression in the VMHdm where PACAP+ neurons are enriched. Data are expressed as mean ± SEM. Scale bars **a**, 50 μm; **c** left panel scale bar 50 μm, right panel 25 μm. MeA medial amygdala, VMHdm ventromedial hypothalamus dorsomedial nucleus. Source data are provided as a Source Data file.

We further explored the effects of MeA to VMHdm circuit inhibition and found differences in c-Fos regulation in brain areas acting as afferent/efferent inputs to this circuit or as structures receiving collateral projections. The chemogenetic inhibition resulted in lower activations of PACAP+ neurons in the anterior hypothalamus (*t*(10) = −2.614, *P* = 0.026) and bed nucleus of the stria terminalis medial anterior part (*t*(10) = −2.287, *P* = 0.045). Also, lower overall c-Fos counts were observed in the anterior hypothalamus (*t*(10) = −2.547, *P* = 0.029), bed nucleus of the stria terminalis medial anterior part (*t*(10) = −2.526, *P* = 0.030), Prelimbic Cortex (t(10) = −2.783, *P* = 0.019) (Supplementary Fig. 7a–c). Despite our approach was not specific to PACAP+ neurons, we observed that around 80% of mCherry+ neurons had PACAP+ immunolabeling (Fig. 5a, b). Moreover, there was prominent VGLUT2 expression in the VMHdm where PACAP+ neurons were located (Fig. 5c). Of note, we did not find significant differences in PACAP expression in the VMHdm between basal and 90 min post-IMO females (*t*(9) = 0.642, *P* = 0.537) or males (*t*(5.153) = −1.114, *P* = 0.315). No effects for sex or stress on PACAP expression were found in the VMHdm (stress * sex, *F*(1,18) = 1.665, *P* = 0.213; sex, *F*(1,18) = 0.013, *P* = 0.909; stress, *F*(1,18) = 0.130, *P* = 0.722) or MeA (stress * sex, *F*(1,19) < 0.001, *P* = 0.996; sex, *F*(1,19) = 0.477, *P* = 0.512; stress, *F*(1,19) = 2.548, *P* = 0.127) (Supplementary Fig. 7d–f). In sum, these experiments showed that PACAP dynamics in the VMHdm after trauma in mice are influenced by the activity of neurons projecting from the MeA. Their inhibition rescues the post-traumatic FE-deficient phenotype and it is associated with lower PACAP levels in MeA and VMHdm, enhanced activity of the MeA, and decreased activity in structures related to the threat detection system.

### No association between the menstrual cycle phase and post-traumatic symptoms after a traumatic experience in women

**Fig. 6:**
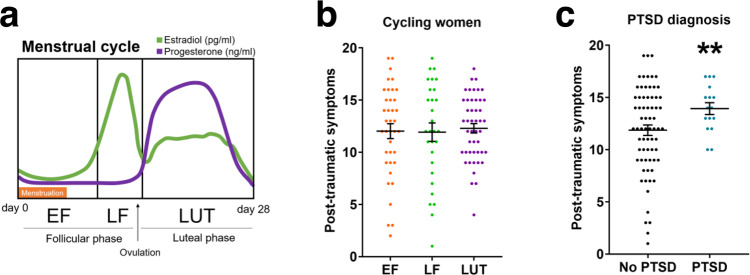
Post-traumatic symptoms and menstrual cycle phases. **a** Menstrual cycle and related hormonal levels in women. **b** Post-traumatic symptom scores of women at 3 weeks post trauma analyzed by menstrual cycle phase at trauma (EF: *n* = 38, LF: *n* = 29, LUT: *n* = 47) **c**, or PTSD diagnosis within the first year of follow-up (PTSD: *n* = 16, No PTSD: *n* = 69) (*P* = 0.009). Data are expressed as mean ± SEM. **P ≤ 0.01. In **b**, the main effect menstrual cycle was analyzed in a one-way ANOVA. In **c**, post-traumatic symptoms were analyzed using a two-tailed *t* test. EF early follicular, LF late follicular, LUT luteal phase, PTSD post-traumatic stress disorder. Source data are provided as a Source Data file.

We studied a cohort of women attending the Emergency Department at the Hospital Clínic de Barcelona (Supplementary Table 1 for demographics) shortly after suffering sexual abuse (<96 h) and documented their menstrual cycle phase at the time of trauma (early follicular, late follicular, luteal) (Fig. 6a). We assessed post-traumatic symptoms 3 weeks after trauma with the Acute Stress Disorder Interview, a clinical tool previously validated for post-traumatic symptom assessment^51^. Our results showed that all women had similar post-traumatic symptom scores regardless of their menstrual cycle phase at trauma (*F*(2,111) = 0.088, *P* = 0.915) (Fig. 6b). The lifespan distribution of PTSD prevalence suggests that age can moderate post-traumatic symptoms^52^. The mean age in our sample was 30 ± 10.4 years and it was not correlated with post-traumatic symptom scores (*r* = 0.001, *P* = 0.987) (Supplementary Fig. 8a). Furthermore, we observed that some women were not conscious during trauma which could have affected their ability to process the event. In our study, there was no significant difference in post-traumatic symptom severity between women that were conscious during trauma compared to women that were not-conscious women (*t*(152) = 1.949, *P* = 0.053) (Supplementary Fig. 8b). We repeated the analysis controlling for consciousness status and the effect of the menstrual cycle phase and found no significant effect (*F*(2,96) = 0.226, *P* = 0.798).

In our sample, 17.6% of women had a history of previous sexual abuse in adulthood (PSAA) and 20.5% history of previous aggression in adulthood (PAA) (Supplementary Table 2). However, for these women their mean post-traumatic symptom scores did not differ compared with women without a history of PSAA (*U* = 1790.500, *P* = 0.635) or PAA (*U* = 2350.000, *P* = 0.655). We analyzed separately to take into account childhood trauma history and found similar symptom scores in women with a history of sexual (CSA; *U* = 2473.000, *P* = 0.366) or physical abuse (CPA; *U* = 2586.000, *P* = 0.144), but women who had experienced emotional abuse had greater symptom scores (CEA; *U* = 2515.000, *P* = 0.016). To know if these variables were interacting with the menstrual cycle phase, we performed additional independent analysis introducing them as between-subject factors. Mean post-traumatic symptom scores were similar regardless of menstrual cycle phase and history of PSAA (*F*(2,91) = 0.449, *P* = 0.639), PAA (*F*(2,101) = 0.248, *P* = 0.781), CEA (*F*(2,85) = 1.672, *P* = 0.194) or CPA (*F*(2,87) = 0.211, *P* = 0.811). A cycle * CSA interaction was observed given by different scores in women in EF (*F*(2,85) = 4.481, *P* = 0.014; EF no CSA vs EF CSA, *P* = 0.033).

Then, we analyzed for post-traumatic sub-symptoms (dissociation, re-experiencing, avoidance, hyperactivation) and observed that the menstrual cycle phase at trauma was not a factor related to the severity of post-traumatic sub-symptoms as measured in the cohort (Supplementary Fig. 8c). However, women that were conscious during the sexual assault had greater severity of re-experiencing symptoms (*U* = 1708.000, *P* = 0.001) (Supplementary Fig. 8d). In addition, participants that were diagnosed with PTSD in medical follow-ups (median 321 days, range 46–1339) (Supplementary Table 3) had greater post-traumatic symptom severity in the assessment at the 3rd week post trauma (*t*(41.871) = 2.738, *P* = 0.009) (Fig. 6c). In these women, the menstrual cycle phase was not associated with PTSD diagnosis, 4 of 20 women in the early follicular phase were diagnosed with PTSD, 1 of 15 in late follicular phase, and 5 of 32 in luteal phase (*P* = 0.548, two-sided Fisher’s exact test). These findings suggest that the menstrual cycle phase at the moment of trauma is not a factor associated with the severity of post-traumatic symptoms.

### *ADCYAP1R1* genetic variants are related to fear extinction deficits in traumatized women

PACAP-PAC1R is known to be regulated after stress exposure and prior work suggested that traumatized women carrying a risk genotype in the *ADCYAP1R1* rs2267735 SNP have greater post-traumatic symptoms^15,53^. More specifically, the homozygous “CC” allele has been linked to increased total PTSD symptom severity^15^, elevated hyperarousal^15^, and greater emotional numbing^54^. Further, a recent meta-analysis revealed that the “C” allele of rs2267735 conferred a significant risk for PTSD in a combined dataset comprised of both sexes; the risk that only remained in a female subset when each sex was examined separately^55^. Taken together, the presence of the “CC” genotype together with low estradiol levels, decreased *ADCYAP1R1* expression, and exposure to traumatic stress can produce a phenotype, including both higher PTSD symptoms and elevated conditioned fear responses^56^. For this reason, the “CC” allele has been identified as a “risk allele” within this pathway in women as compared to “G” carriers who have been repeatedly classified as “low-risk”^55,57^. Our prior studies of extinction in this population have indicated that lower estrogen levels were associated with higher startle responses during the early phase of extinction, which was in turn associated with symptoms of PTSD^58–60^. However, we had not examined PAC1R genotype effects on FE.

**Fig. 7:**
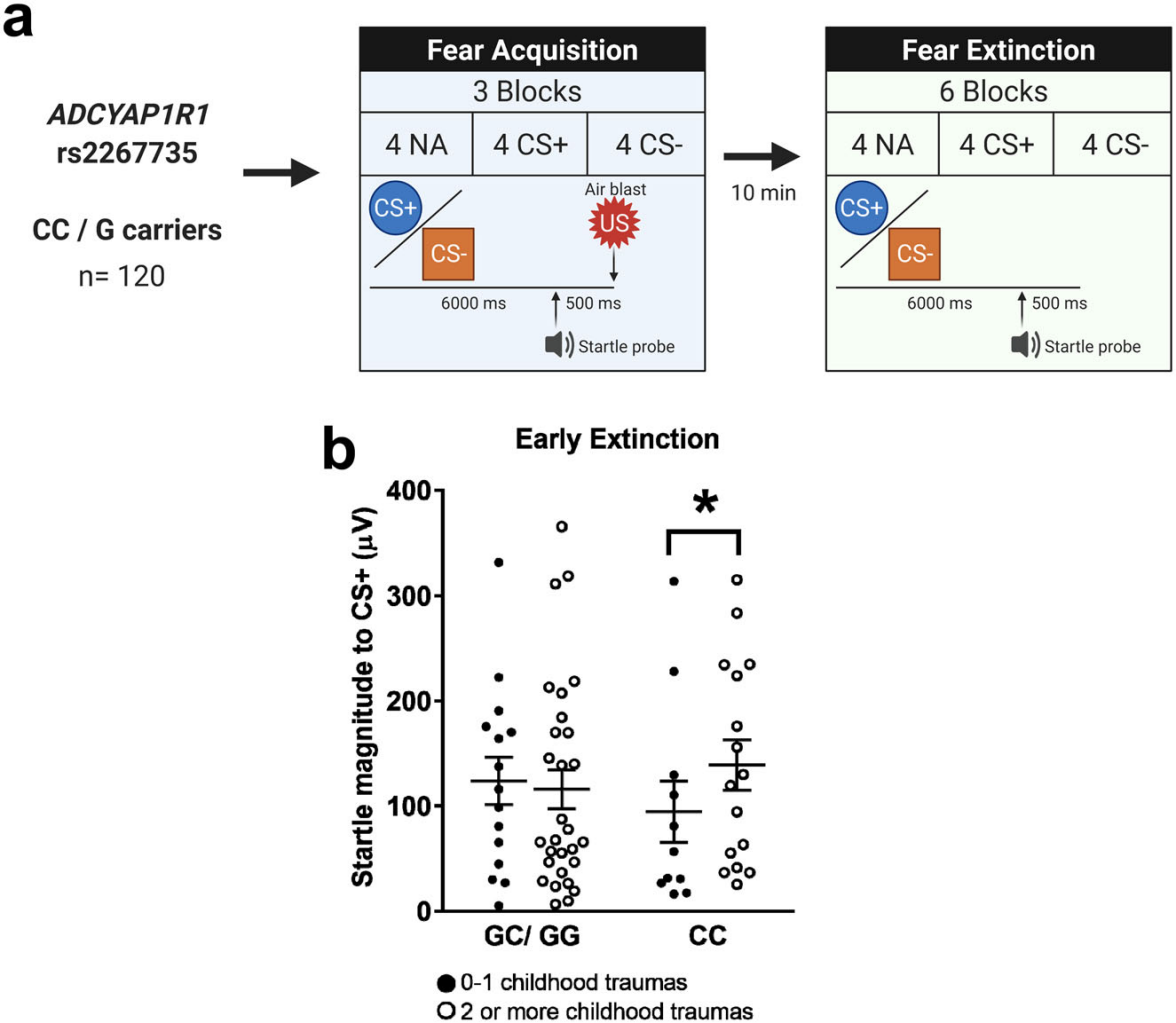
*ADCYAP1R1* rs2267735 genotype effects during early FE in highly traumatized individuals. **a** Methods used for the assessment of the influence of rs2267735 genotype over FE (*n* = 71, 49). **b** Fear-potentiated startle magnitude of cycling women (≤40 years old) with CC or GC/GG genotypes during early FE and its relation to childhood trauma exposure (*n* = 71) (*P* = 0.030). Analyses for >40 years old (*n* = 49) are shown in Supplementary Fig. 9c. Data are means ± SEM. *P ≤ 0.05. Fear-potentiated startle data in **b** were analyzed with an ANCOVA with extinction as repeated measures variable, age, genotype, and childhood trauma as between-subjects variables. Covariates included GWAS principal components, total lifetime trauma, baseline startle. None of the CS+ presentations was paired with an US during the FE phase. CS+ reinforced conditioned stimulus, CS- non-reinforced conditioned stimulus, NA noise probe alone, US unconditioned stimulus. Source data are provided as a Source Data file.

Therefore, we studied fear-potentiated startle during extinction in another cohort of women from the Grady Trauma Project. Care was taken to ensure that participants’ trauma was not exacerbated by their participation, as detailed in the Methods section (see Supplementary Table 4 for demographics) (Fig. 7a). Women above 40 years old have a cumulative incidence of irregular cycles starting from 10% at 40 to 80% at 50 (see ref. ^61^). Hence, we divided women into two age groups, ≤40 years (*n* = 71) and over 41 years (*n* = 49) and focused our analyses on the younger age group. Of note, in the experiments for Fig. 6b, c, we removed women with irregular cycles from the analyses. In an analysis of covariance (ANCOVA), we analyzed extinction as a repeated measures variable (two levels: early, late), and age and genotype as between-group variables. As in our prior studies where we found interactions with childhood trauma^62,63^, we categorized women as having experienced 0–1 types of childhood abuse or 2+ types of childhood abuse from the Childhood Trauma Questionnaire. We also entered the top two GWAS principle components, total lifetime trauma, and baseline startle as covariates in the analyses. At the end of fear conditioning, startle responses were greater to CS+ compared to CS-, (*F*(1,111) = 9.082, *P* = 0.003), but no interaction with age, childhood trauma, or genotype (*P* = 0.937), showing that FC was successful but did not differ between groups. There was a significant reduction of startle from early to late extinction (*F*(1,108) = 10.640, *P* = 0.001), as well as a significant three-way interaction of age * genotype * childhood trauma on startle (*F*(1,108) = 4.131, *P* = 0.045) even when adjusting for covariates (Supplementary Fig. 9a–c). Follow-up analyses with the same control variables found that in women ≤40 years, genotype and childhood trauma had a two-way interaction on startle during early extinction, with the CC genotype associated with a higher startle in those with two or more childhood trauma exposures (*F*(1,64) = 4.951, *P* = 0.030) (Fig. 7b). Notably, we did not see the same associations in the older age category (*F*(1,64) = 1.514, *P* = 0.225) (Supplementary Fig. 9c). These results suggest that women that had experienced a high level of childhood trauma load, and with a risk genotype in the *ADCYAP1R1* rs2267735 SNP have impairments in FE.

## Discussion

In this study, we found that female mice subjected to an acute traumatic stressor developed long-lasting alterations of FE that were unrelated to the phase of the estrous cycle at the moment of trauma. In these mice, IMO exposure upregulated *Adcyap1-Adcyap1r1* transcripts in the amygdala and hypothalamus and changed PACAP+ neuronal activation in MeA and VMHdm. We found that PACAP in the VMHdm is relevant for stress responses and that the inhibition of MeA to VMHdm circuit during trauma alters PACAP short-term dynamics and rescues the IMO-induced FE impairments. In addition, in a cohort of women who experienced sexual assault we observed no association for the menstrual cycle phase at trauma with post-traumatic symptom scores at 3 weeks post trauma. Further, women with a history of multiple traumas and a risk genotype in the *ADCYAP1R1* rs2267735 SNP had impairments in FE.

Our data showed that IMO is an acute and intense emotional stressor that produces deficits in FE that are coupled with physiological adaptations to trauma as evidenced by a decreased weight gain in female mice. Female mice exposed to IMO during proestrus or metestrus had similar increased freezing levels during FE and similar post-stress corticosterone levels. However, mice in proestrus and metestrus presented differences in the regulation of progesterone, testosterone, and estradiol after stress. These differences have been previously characterized for progesterone and they have shown to vary depending on the type of stressor^64^. To note, the effects of IMO over FE and weight gain in females with cycle monitorization were attenuated compared to females without cycle monitorization. This attenuation may be related to their exposure to repeated manipulations for cytology assessment and the habituation of their stress response, although the exact mechanisms remain to be discovered^65–67^.

The menstrual cycle is known to influence performance in emotional tasks. For example, menstrual phases with high estradiol levels are related to better FE consolidation, less subjective distress, and attenuations in the activity of cortical and subcortical arousal regions^30,68,69^. However, research focused specifically on the effects of traumatic injury found that trauma experienced in distinct phases of the menstrual cycle was associated to similar PTSD symptom scores, but women experiencing trauma in the mid-luteal phase reported having more flashbacks^11^. Our findings are in line with this study in the sense that the menstrual cycle phase at trauma was not a factor associated with overall greater post-traumatic symptomatology. However, we did not find greater re-experiencing symptoms associated with any menstrual cycle phase after performing a more detailed segmentation into menstrual cycle phases and controlling for irregular cycles and oral contraceptive use.

Hence, the menstrual cycle phase and hormonal levels may not be factors influencing the encoding of traumatic memories, rather the recollection of aversive memories or the performance during mildly stressful tasks. Research studies that have found an association for the luteal phase with greater flashback memories perform the encoding and retrieval of memories during the same luteal phase or during the early follicular phase (low hormones)^11,70,71^. Indeed, this memory retrieval effect was also found in women with PTSD that were evaluated during the early follicular phase and who showed greater avoidance, fear-related and overall symptom severity compared to women evaluated during the mid-luteal phase^10^. Altogether our results suggest that the hormonal cycle phase at trauma is not associated with the further development of a more severe post-traumatic phenotype. Since our extinction paradigm in both mice and women is unrelated to the trauma, our conclusions about the lack of effects of the cycle during trauma do not necessarily predict whether extinction-based therapies for the memories acquired during the trauma itself would be different based on the cycle at trauma.

Previous research showed that PACAP-PAC1R function is altered in female clinical populations but its mechanistic contribution to disease remains elusive^15,16^. PACAP is implicated in central and sympathoadrenal responses to emotional stressors^72^ and *Adcyap1r1* transcripts increase after FC in both male and female mice^15,16^. Here, we found that female mice exposed to IMO upregulated *Adcyap1-Adcyap1r1* mRNA in the amygdala and hypothalamus after a FE session when compared to animals receiving compensatory handling. Thus, exposure to traumatic stress can prime PACAP-PAC1R for overactivation under a FE task. In addition, in our cohort of highly traumatized women the CC genotype in *ADCYAP1R1* rs2267735, which has been associated with vulnerability^15,17^, produced impairments in the early phases of FE. Notably, this risk genotype is associated with lower *ADCYAP1R1* expression and decreased function of estradiol as an adaptive stress response^16^. The alterations in FE were clear in women ≤40 years old but only trend level in older women, an effect that may be related to lower estradiol levels with increasing age, but also to other age-related variables.

Concerning the neural mechanisms of action, we observed that IMO induced impairments in FE, deficits in MeA function, and increased the activation of PACAP+ neurons in the VMHdm. Brain circuits between the amygdala–hypothalamus are involved in stress and threat processing. In mice, the MeA is strongly activated by IMO and modulates behavioral and hypothalamic neuroendocrine responses^43,73^, while the stimulation of VMH can induce fear responses like immobility or avoidance^74^. Together, the MeA-VMH are part of a medial hypothalamic defensive system that is implicated in the generation of innate and conditioned defensive responses to conspecific/predator cues^75–77^. Since the MeA is a major source of input to the medial hypothalamic defensive circuit that is strongly activated by IMO, we hypothesized that it could be relaying signals to the VMHdm to increase PACAP function that further facilitated VMHdm activation. In our results, a single traumatic exposure in female mice induced functional changes in the hypothalamus, through the MeA to VMHdm projections, that were related to deficits in FE but not fear learning. These changes in FE may be related to the functional restructuration of basal behavioral circuits that facilitate the organism's survival or coping mechanisms.

We also found a neuronal signature of trauma after FE sessions, the lower levels of PACAP-c-Fos+ neurons in the MeA after FE4 are related to a previously unknown process of neuronal adaptation after a traumatic stressor. The reasons for this decrease in PACAP-c-Fos+ expression weeks after IMO exposure, once the post-traumatic phenotype is established, could be simply reflecting the neuronal signature of trauma. However, PACAP-c-Fos+ expression in the MeA is likely necessary for normal FE to occur. Future studies focused on the effects of trauma over FE should be able to clarify this. The VMH displays high interconnectivity with hypothalamic and diencephalic structures playing a central role in homeostasis by regulating mating, feeding, aggression, and metabolism^75,78^. Previous research showed that excitatory activity in the VMHdm increases defensive responses through “one-to-many” wiring configurations^74,79^. Moreover, a strong activation in the VMH leads to persistent activity and persistent defensive behaviors that rely on neurotransmitter release and recurrent excitatory networks^45^. Notably, a study that used a model of foot shocks as a stressor in males found that MeA-VMH synapses were potentiated after the shocks and related to stress-induced increases in aggression^80^. Our findings are in line with these studies and suggest that the VMHdm in female mice may have undergone synaptic potentiation after IMO and promoted an aversive internal state that facilitated coping with threatening stimuli but to the detriment of FE.

We speculate that the neuronal ensembles representing sensory and neuroendocrine aspects of the acute stress response were altered by the chemogenetic inhibition of MeA to VMHdm neurons. The increase in c-Fos+ neurons in the MeA after chemogenetic inhibition may be secondary to these neurons being embedded in intra-amygdala inhibitory circuits or feedback loops within brain-wide stress-response circuits. Both, the amygdala ensembles, and intra-amygdala inhibitory circuits are crucial for fear learning and fear extinction^81–83^. In our results, we observed that the manipulation of the MeA to VMHdm circuit influenced brain-wide neuronal activity in a larger threat detection system. This suggests that the information processed by the MeA and relayed to the VMHdm or other structures is crucial for processing sensory and neuroendocrine aspects of trauma. In addition, the specific inhibition of the MeA to VMHdm circuit resulted in short-term changes in PACAP dynamics after trauma. Higher PACAP levels in the MeA and VMHdm of control animals shortly after IMO were related to the appearance of a FE-deficient post-traumatic behavioral phenotype. Also, there is evidence demonstrating that PACAP signaling can increase glutamatergic function through NMDA-dependent mechanisms in the VMH^84,85^. These findings suggest a glutamatergic role in the VMHdm after exposure to traumatic stress that is relevant in our model. Future studies may delineate if trauma-induced changes in VMH function are dependent on this mechanism.

Classical FE studies have shown that amygdala–hippocampal–prefrontal circuits are the main drivers of FE, but stress exposure can promote structural and functional changes^42,86^. These FE circuits are embedded into a larger defensive circuitry which ultimately executes appropriate behaviors in the face of a threat. The VMH is capable of promoting aversive internal states and is well-positioned to mediate the integration of stress and fear-related behaviors through its direct connections with the BLA, BNST, PAG, or the paraventricular thalamus^74,87–89^. Interestingly, some evidence shows a role for the VMH in humans for the induction of panic attacks^90^. Given that most of these results come from studies that have used male rodents, they should be carefully considered when translating findings to female rodents or humans. In our paradigm, we were mainly engaging the VMHdm and the defensive parts of the amygdala which are reported to work similarly in males and females^74,91^. However, some subnuclei in the MeA and VMH are sex-dimorphic, influenced by sex hormones, and regulate reproductive and defensive responses to conspecifics^77,92^. Future studies should consider the type of stressor used when studying sex differences. In humans, traumatic stress secondary to interpersonal violence or sexual abuse has a large pathogenic potential and is one of the most common sources of trauma in the population^93–95^. In sum, our results shed light on the role of PACAP-PAC1R in VMH for acute stress processing in female mice and show that the MeA to VMHdm circuit regulates PACAP short-term dynamics after an intense acute stressor and the appearance of a FE-deficient post-traumatic behavioral phenotype.

These findings come along with limitations. In the mouse study we only selected the two most representative and classically studied phases of the estrous cycle: proestrus (high hormones), metestrus (low hormones), it remains to be determined if these findings extend to each phase of the estrous cycle. The chemogenetic manipulation we used was successful for rescuing trauma-induced impairments in FE and PACAP regulation, although it was not specific for PACAP neurons.

In our study of women who had experienced trauma, we attempted to determine the menstrual phase at time of trauma carefully as possible, using a method based on the last menstrual period date and reported cycle regularity. However, for this calculation, we had to exclude a proportion of young women with irregular cycles or women in and near menopause. A further limitation is that we had a high proportion of missing information regarding the history of previous trauma exposure which could have affected the analyses on the moderation of menstrual cycle*trauma history. Unlike our results on post-traumatic symptom severity, our analyses on PTSD diagnosis and menstrual cycle phase rely on a small sample size and should be interpreted accordingly. In humans, experiencing violence and aggression have a high potential for leading to mental health conditions such as PTSD. Future studies focused on testing specifically for memories directly related to the trauma may inform if the menstrual cycle stage should be taken into account for people receiving extinction-based therapies. Importantly, these studies must ensure that the participants are not retraumatized by their participation.

In sum, we demonstrate that the menstrual cycle stage at the moment of traumatic stress is not associated with the severity in post-traumatic symptoms. Furthermore, in a mouse model, stage of estrous cycle at time of modeling trauma does not affect PTSD-like behavior. We also found that the PACAP-PAC1R system is important for fear extinction in highly traumatized women and a PTSD-like model in female mice. Additionally, our study discovered that high levels of PACAP in the VMHdm lead to vulnerability to the negative consequences of stress whereas low levels of PACAP may result in resilience. Growing evidence points at the PACAP-PAC1R system as crucial for stress processing, with its dysfunction being associated with worse outcomes in women. Further studies may potentially find therapeutic interventions involving the PACAP-PAC1R system. However, one of the problems, from a pharmacology point of view, is the widespread expression of the PACAP-PAC1R system. We have previously proposed that targeting specific neuronal populations that colocalize with PACAP in emotional areas of the brain could offer advances to the current understanding of fear and stress processing^37^.

## Methods

### Mice

#### Ethics and biosecurity protocols for mice experiments

Ethics protocols were approved for the experiments in mice ref. CEEAH 3603 and biosecurity protocols 345-16 and 407-17 by the Committee of Ethics of the Universitat Autònoma de Barcelona and the Generalitat de Catalunya. Institutional Animal Care and Use Committee (IACUC) protocol for the mice was McLean-based, and 2017N000228. Experiments were carried out following the European Communities Council Directive (2010-63-UE) and Spanish legislation (RD 53/2013).

#### Animals

Behavioral experiments were performed on adult (8–12-week-old) wild-type C57BL/6J male and female mice (Charles River, Spain). Antibody-validation experiments from Supplementary Fig. 4a, b were performed on 6-month-old PACAP^flox/flox^ females. Mice undergoing surgery were housed in pairs, the rest in groups of four and kept under standard conditions of temperature (22 ± 1 °C) and humidity (~40%) in a 12 h light/12 h dark schedule (lights on at 8:00 h), with ad libitum food and water intake^96^. Behavioral procedures and pharmacological manipulations began early in the light phase of the cycle. Male and female mice were housed separately in the same room.

#### Immobilization stress in mice

IMO was performed as previously described^18^. Briefly, IMO was conducted in a room separate from the housing and behavioral paradigms. Each animal was immobilized by gently restraining its four limbs in a prone position to metal arms attached to a wooden board for 2 h (Panlab-Harvard apparatus, Spain). All animals in the same cage received the same treatment—either IMO or compensatory handling. Handling lasted ~5 min per mouse and consisted of letting the animal walk on top of their home cage and in the hands of the experimenter wearing latex gloves. After stress exposure or compensatory handling, animals were returned to their home cage where they remained undisturbed until fear training. FC testing started 6 days after IMO/ handling. Of note, 2 days before IMO/handling exposure mice were habituated to the context of the FC chambers for 5 min.

#### Cued-fear conditioning and fear extinction in mice

FC and FE procedures were carried out with a computerized StartFear system (Panlab-Harvard, Spain) as previously described^26^. Tones and shocks were delivered and controlled using Freezing v1.3.04 software (Panlab-Harvard apparatus, Spain). The fear chamber consisted of a black methacrylate box with a transparent front door (25 × 25 × 25 cm) inside a sound-attenuating chamber (67 × 53 × 55 cm). The same boxes were used for FC and FE. Animals were habituated to the fear chambers for 5 min/day for two consecutive days before IMO. During the cue-dependent FC, mice were placed in the fear chambers for 5 min and then received five trials of a tone (30 s, 6 kHz, 75 dB), as the conditioned stimulus (CS), that co-terminated with a foot-shock (1 s, 0.3 mA), as the unconditioned stimulus (US). The intertrial interval (ITI) was 3 min, and 3 additional min followed the last trial. The FE tests were performed 4 times in consecutive days (FE1, FE2, FE3, FE4) starting 24 h after FC. For FE, mice were placed in the fear chambers for 5 min and then exposed to 15 trials of the 30 s CS tone alone (cued fear) with a 30 s of ITI interval. An additional 30 s interval followed the last trial of FE. Freezing behavior, a rodent´s natural response to fear defined as the absence of movement except for respiration, was scored by a high sensitivity weight transducer system located at the bottom of the experimental chambers which records and analyses the signal generated by the movement of the animal. Freezing was scored when mice remained immobile for more than 1 s and episodes were averaged in 30 s slots using Freezing v1.3.04 software (Panlab-Harvard apparatus, Spain). Different contexts were used for FC and FE tests. Habituation and FC context consisted of a yellow light source (~10 lux), a grid floor of 25 bars (3 mm Ø and 10 mm between bars), the background noise of 60 dB produced by a ventilation fan, and a solution of ethanol 70% as odor. FE context consisted of a red-light source (~10 lux), a gray plexiglass floor covering the bars, no background noise, and CR36 (bronopol 0.26%, benzalkonium chloride 0.08%, and isopropyl alcohol 41%) (José Collado, Spain) as odor. The chambers were carefully cleaned with soapy water before and after each mouse. Also, different routes were used to transport them from their home cages to the testing room on FC and FE days.

#### Vaginal smear cytologies in mice

The estrous cycle was monitored in a cohort of naturally cycling female mice subjected to IMO or compensatory handling. Vaginal cytologies were adapted from known protocols^97^, and performed at 9:00–11:00 h. Subsequent behavioral procedures or sample collection took place after 2 h. Vaginal smear cytologies were obtained for 8–12 days before testing to ensure estrous cycle regularity. A vaginal lavage was performed with 10 µl of standard NaCl 0.9% solution using five flushes at the entrance of the vaginal aperture. The vaginal lavage was then placed on an adhesion slide, dried, stained with 0.1% Cresyl violet Acetate (Sigma-Aldrich, Spain), washed twice in distilled water, and read in brightfield microscopy at ×10 or ×20. The estrous cycle phase was determined by the proportions of cells in the cytology (cornified, nucleated, leukocytes) as described in ref. ^67^. Proestrus is characterized by a predominance of nucleated cells with few leukocytes or cornified cells; estrous consists mostly of cornified cells; metestrus has a predominance of cornified cells with leukocytes and diestrus is characterized by a predominance of leukocytes. Estrous phase monitoring was carried out from 3 to 9 consecutive days (until the desired phase was identified). Only animals in proestrus or metestrus were selected.

#### Reverse transcription and quantitative PCR

A separate cohort of female mice was sacrificed 30 min after fear expression (FE1). Brains were immediately fresh frozen on isopentane cooled with dry ice and stored at −80 °C. Bilateral tissue punches from the prefrontal cortex, amygdala, hypothalamus, and periaqueductal gray were performed based on the Mouse Brain Atlas by Paxinos^98^ and individually stored. Total RNA was isolated and purified from the tissue with Maxwell RSC simplyRNA Tissue Kit #AS1390 (Promega Biotech Ibérica, Spain)^99^. Gene expression changes were detected by relative quantitative reverse transcription PCR FAST 7500 (Applied Biosystems, USA). cDNA was obtained by reverse transcription using the High-Capacity cDNA Reverse Transcription Kit #4368814 (Thermo Fisher, Spain) according to the manufacturer’s instructions. TaqMan assays (Applied Biosystems, USA) were used to quantify the expression of *Adcyap1* Mm00437433_m1 (#4331182) and *Adcyap1r1* Mm01326453_m1 (#4331182) normalized to mouse *Gapdh* Mm99999915_g1 (#4352932E, glyceraldehyde-3-phosphate dehydrogenase). Statistics are computed with ddCT, graphics are represented as fold changes obtained with the 2^−ΔΔCt^ method^37^.

#### Immunohistochemistry

General procedure: A group of female mice was transcardially perfused with 4% paraformaldehyde (PFA) (Casa Álvarez, Spain) 90 min after the last behavioral procedure. Brains were post-fixed in PFA for 24 h (4 °C) after which they were rinsed (3 ×15 min) with Sorenson’s sodium phosphate buffer (PB) 0.1 M consisting of 10.9 g/l sodium phosphate dibasic (Sigma-Aldrich, Spain), 3.2 g/l sodium phosphate monobasic (Sigma-Aldrich, Spain). Further, they were transferred into 30% Sucrose (Sigma-Aldrich, Spain) in PB for 48–72 h at 4 °C. After this, brains were frozen in Isopentane (Sigma-Aldrich, Spain) cooled with dry ice, and stored at −80 °C until sectioning. In all, 30-μm coronal sections were cut on a cryostat and stored at −20 °C in an anti-freeze solution (30% ethylene glycol (Sigma-Aldrich, Spain), 20% glycerol (Sigma-Aldrich, Spain) in 0.1 M phosphate buffer)). For immunohistochemistry, free-floating slices were rinsed thoroughly in KPBS and then incubated for 60 min at room temperature in blocking buffer (5% Donkey Serum and 0.4% Triton-X (Sigma-Aldrich, Spain) in potassium phosphate-buffered saline (KPBS)). Primary antibodies were diluted in 0.4% Triton-X in KPBS and incubated in agitation at 4 °C overnight. The next day, after three rinses in KPBS, slices were incubated in agitation with secondary antibodies in 0.4% Triton-X in KPBS at room temperature for 2 h. After incubation, slides were rinsed in KPBS and DAPI 1:10,000 (Sigma-Aldrich, Spain) to stain cell nuclei. Immediately after, they were mounted on glass slides and coated with Fluoromount-G Mounting Medium (Thermo Fisher, Spain). The following primary antibodies were used: anti-c-Fos (1:500, ab6167) (Abcam, UK), anti-FosB [83B1138] (1:2000, ab11959) (Abcam, UK), anti-PACAP-38 (1:1000, T-4469) (Peninsula Laboratories, USA), anti-PAC1R (1:100, ab54980) (Abcam, UK) and mouse monoclonal anti-VGLUT2 [8G9.2] (1:300, ab79157) (Abcam, UK). As secondary antibodies: AlexaFluor-647 donkey anti-sheep (1:500, A-21448) (Fisher Scientific, Spain), Rhodamine-Red donkey anti-mouse (1:500, 715-295-151) (Jackson Antibodies, UK), and AlexaFluor-488 donkey anti-rabbit (1:1000, 711-545-152) (Jackson Antibodies, UK). For PACAP antibody-validation free-floating slices between bregma −1.1 and −1.8 mm were incubated with primary antibody anti-PACAP-38 (1:5000, T-4469) (Peninsula lab, USA) and secondary antibody Alexa fluor 647 donkey anti-rabbit (1:1000, A-31573) (Invitrogen, USA).

Image acquisition: The following structures were analyzed according to the Mouse Brain Atlas (Paxinos and Franklin, 2001): basolateral amygdala (BLA), bed nucleus of the stria terminalis anterolateral (BSTal), dorsal CA1 hippocampal region (dCA1), dorsal CA3 hippocampal region (dCA3), central amygdala (CeA), cingulate cortex (Cg), dorsal dentate gyrus hippocampal region (dDG), ventral dentate gyrus hippocampal region (pDG), dorsomedial hypothalamus (DMH), infralimbic cortex (IL), insular cortex (Ins), medial amygdala (MeA), dorsolateral periaqueductal gray (PAGdl), ventrolateral periaqueductal gray (PAGvl), prelimbic cortex (Prl), ventral lateral septum (vLS), ventromedial hypothalamus dorsomedial nucleus (VMHdm). Immunofluorescence images were captured using a Zeiss LSM 700 Confocal microscope and Zen2010 software (Zeiss, Spain) under a dry 20× magnification lens. Five to six images per structure per animal were acquired using four different laser lines (405, 488, 555, and 639 nm). Two-dimensional overview pictures were obtained for the colocalization experiment (8 bit, 1024 × 1024 pixels). For PACAP quantification experiments three-dimensional *Z*-stacks were acquired in 1.5 µm thin optical planes along six planes (9 µm). Two-dimensional overview pictures were obtained by *z* projection (16 bit, 1024 × 1024 pixels) and further analyzed blind to the experimental group in Image J software (Fiji v.1.53c, Rasband W, NIH, USA). For the mosaics, *Z*-stacks (1.51 μm/interval) were acquired using a Leica SP5 confocal microscope (Leica, Spain) with a dry ×20 magnification lens at ×0.5 zoom and processed using the software Leica Application Suite Advance Fluorescence (LAS AF) Version 2.7.3.9727 (Leica Microsystems, Germany). A two-dimensional overview picture was obtained from the maximum intensity projection made in Image J software. For PACAP antibody validation, images were taken using the Echo Revolve fluorescent microscope (Echo, USA), merged using Adobe photoshop, and processed with Image J.

Quantification: For analyses, the average background signal for each picture and channel was manually measured and subtracted, a Gaussian filter (sigma 1) was used to improve signal detection, thresholds were used to segment the images and to create binary masks, masks were visually inspected and corrected. For colocalization experiments, a DAPI mask was overlaid on each channel’s mask with the LungJ plugin and colocalized cells were automatically counted using the analyze particles plugin. Automatic and manual counts were compared in a subset of pictures obtaining a high correlation (*r* > 0.95). Labeled neurons were calculated as the average number of cells per mm^2^. Colocalization counts were normalized to DAPI for each picture and averaged for each mouse. Colocalization results are shown in relation to controls. For PACAP signal quantification, masks for each channel were overlaid to measure the integrated density (intDen = area × mean intensity). Protein levels were normalized to DAPI for each picture and averaged for each mouse.

#### Surgeries and viral vector microinjection

Ovariectomy surgery: Ovariectomy surgeries were performed in a subset of females to use their serum to make the standard curve preparation for the ELISA estradiol kit. Mice were anesthetized with 4% isoflurane for induction, and 2–3% for maintenance, in oxygen at a constant rate of 1.5 l/min. After skin shave and disinfection with EtOH 95% and iodine povidone 10%, ovariectomies were performed making a bilateral incision on the back of the animal, 1 cm lateral to the midline and right over the back limbs line. Adipose tissue was extracted, and the ovary was localized and isolated making a knot with sterile absorbable suture thread (Centauro, Spain) around the oviduct. The ovary was extirpated and the adipose tissue, containing the rest of the oviduct, was returned to the abdominal cavity. The muscle was sewed with sterile absorbable thread and the skin was sewed using sterile silk suture (Centauro, Spain). Mice remained undisturbed for 8 weeks until trunk blood was collected to avoid any effect of previous estradiol.

Stereotaxic surgery: Mice were anesthetized with 4% isoflurane for induction, and 2–3% for maintenance, in oxygen at a constant rate of 1.5 l/min. Animals were placed in the stereotaxic frame (Harvard-Panlab, Spain) and aligned in the antero-posterior (AP) and lateromedial (LM) planes. The coordinates for AAV injection in relation to bregma according to Mouse Brain Atlas by Paxinos and Franklin^98^ were (MeA) AP −1.3 mm, ML ±2.0 mm, DV −5.25 mm; (VMHdm) AP −1.3 mm, ML ±0.25 mm, DV −5.25 mm. Animals received bilateral intra-MeA or intra-VMHdm injections of 0.3 µl of virus/side at a constant rate of 0.1 µl/min with a microinfusion pump (Harvard Apparatus, USA). For antibody-validation studies, unilateral infusions of 0.3 µl of virus/side into the (VMHdm) AP −1.3 mm, ML 0.3 mm, DV −5.25 mm and (MeA) AP −1.3 mm, ML 2.0 mm, DV −5.25 mm. The matched contralateral region within the same subject was used as the control condition. After infusions, the needle was left in place for an additional period of 10 min to allow the fluid to diffuse and to prevent reflux, then it was slowly withdrawn during 10 additional min. The skin was closed using a 3-0 Polyamide suture.

Viral vectors: The viral vectors used were AAV-hM4Di-DREADD (AAV8-hSyn-DIO-hM4D(Gi)-mCherry, 1.21E  +  13 gc/ml), AAV-control-DREADD (AAV8-hSyn-DIO-mCherry, 1.19E  +  13 gc/ml) from Viral Vector Production Unit of Universitat Autònoma de Barcelona and AAV-retrograde-Cre-EBFP (AAVrg pmSyn1-EBFP-Cre; 6 × 10^12^ vg/ml) from Addgene (viral prep # 51507-AAVrg). To inhibit MeA neurons projecting to the VMHdm, mice received bilateral injections of 0.3 µl of AAV-retrograde-Cre-EBFP into the VMHdm and bilateral injections of 0.3 µl of AAV-hM4Di-DREADD or AAV-control-DREADD in the MeA. To inhibit VMHdm neurons projecting to MeA, mice were injected bilaterally with 0.3 µl of AAV-retrograde-Cre-EBFP into the MeA and 0.3 µl of AAV-hM4Di-DREADD or AAV-control-DREADD in the VMHdm. For antibody validation, animals received unilateral 0.3 µl of AAV9-CMV-eGFP-Cre (105545-AAV9) (Addgene, USA).

Injection verification: To detect AAV8-hSyn-DIO-mCherry or AAV8-hSyn-DIO-mCherry serial coronal sections containing the MeA and VMHdm were visualized directly under a Zeiss LSM 700 confocal microscope and images from representative slices were obtained with a dry ×20 objective at 0.5 zoom. Injection sites were histologically verified by overlapping to standard stereotaxic plates^98^, direct visualization of needle trajectory and detection of EBFP or mCherry expression. Only animals with at least an ipsilateral pair of injections circumscribed to both the MeA and the VMHdm were included in the study.

#### Drugs

For the activation of the inhibitory designer receptors exclusively activated by designer drugs, animals received an intraperitoneal injection of clozapine N-oxide (CNO) (Tocris, UK) in 0.5% DMSO at 1 mg/kg^37^.

#### Steroid determination

For steroid determination trunk blood was collected after decapitation and allowed to clot at 4 °C. Then it was centrifuged (8000×*g*, 15 min, 4 °C) to collect serum and stored at −80 °C until analyzed. Serum levels of testosterone, progesterone, dehydrocorticosterone, and deoxycorticosterone were determined based on previously reported papers^100,101^. Briefly, 20 µl of serum were mixed with 20 µl of labeled internal standard solution. After proteins precipitation with 100 µl of acetonitrile, samples were centrifuged (3000 × *g*, 5 min) and the supernatant was transferred to a clean tube. The mixture was vortexed and transferred to a clean tube. Serum samples underwent a liquid-liquid extraction by the addition of 1 ml of NaCl (saturated solution) and 4 mL of ethyl acetate. Extracts were centrifuged (3000 × *g*, 5 min) and the organic layer was transferred into a clean tube and dried under a nitrogen stream. Dried extracts were reconstituted with 100 µl of methanol and 10 µl were injected into the LC-MS/MS system consisting of an Acquity UPLC system coupled to a triple quadrupole (TQS Micro) mass spectrometer. Steroid detection was performed by selected reaction monitoring (SRM) including two transitions for each analyte. The most specific one was selected for the quantification. Quantification was performed by an external calibration approach using the TargetLynx module of the MassLynx software (Thermo Fisher, Spain). Estradiol was measured with the ELISA kit ES180S-100 (Calbiotech, USA). For standard curve preparation, serum from ovariectomized mice was used adding known concentrations of estradiol (Sigma-Aldrich, Spain): 0, 3, 10, 30, 100, and 300 pg/ml, using denaturalized EtOH (Casa Álvarez, Spain) as a vector for estradiol. Kit instructions were followed as stated, samples were loaded in duplicates and absorbance was read at 450 nm with the microplate reader Varioskan Lux (Thermo Fisher, Spain) controlled with Skanit for microplates v6.0 software (Thermo Fisher, Spain). The average of duplicates was used as estradiol determinations for each sample.

### Humans

#### Participants and ethics statement

For Fig. 6, Supplementary Fig. 8, and Supplementary Tables 1–3, participants (*n* = 293) were recruited from the Emergency Department in the Hospital Clínic de Barcelona, Spain, as part of a specialized protocol that provides first-aid care in sexually abused women. All women attending the ED in the Hospital Clínic de Barcelona were offered medical, psychiatric, and psychosocial counseling regardless of their participation in the study. The first contact occurred in the ED with a quick, multidisciplinary, and coordinated assessment that ensured to avoid revictimization. Throughout this process, the women were accompanied, received medical treatment, and were offered the opportunity to report the offense to the police. At discharge, in-person follow-ups were carried out monthly with telephonic assessments between them. The follow-up aimed to detect and treat any possible effects of trauma, promote treatment adherence, and ensure the person’s return to normal functioning. Inclusion criteria for the study were willingness to participate, being older than 18 years, and being a victim of a recent sexual assault. Exclusion criteria were language barriers, being a tourist, having mental disabilities or active psychosis, and women ceasing to participate in the study before post-traumatic symptom assessment at the 3rd week post trauma. For the final cohort, we additionally excluded women with missing last menstrual period dates, women in menopause, and women with irregular menstrual cycles for a final cohort of 170 participants. Participants did not receive payment as the study was part of their treatment plan. All women signed the informed consent approved by the Ethics Committee of Clinical Research in the Hospital Clínic de Barcelona.

For Fig. 7, Supplementary Fig. 9 and Supplementary Table 4, participants were part of the Grady Trauma Project, which is a group of investigators studying civilian trauma based at Grady Memorial Hospital and Emory University School of Medicine in Atlanta, Georgia. The project focuses on PTSD and the clinical and physiological implications of trauma exposure. Participants (*n* = 55 CC genotype, *n* = 65 G allele carriers) were recruited from the General Medical Clínic, Primary Care, Diabetes, Sickle Cell and OB/GYN, and Main Pharmacy Waiting Rooms at Grady Healthcare System, a publicly funded, not-for-profit healthcare system that serves the low-income and homeless population in downtown Atlanta, a city of ~4 million people. Participants of different races and ethnic groups were included, both males and females. Participants were approached in the waiting room for screening by trained study staff and the study procedures were explained to potential participants^60^. Inclusion criteria were willingness to participate and the ability to understand the informed consent form. Exclusion criteria included: participants with active symptoms of mania, schizophrenia, or other psychoses; participants with current prominent suicidal ideation, intoxicated participants. Participants with special medical conditions that can contribute significantly to psychiatric symptoms, including hypo- or hyperthyroidism, systemic lupus erythematosus, cirrhosis, and dementia. For the startle testing, participants with a positive urine drug test were excluded. All participants were screened for hearing impairments with an audiometer (Grason-Stadler, Model GS1710), and the ones not able to detect tones of 30 dB(A)SPL at frequencies ranging from 250 to 4000 Hz were not included in the study. We balanced three primary concerns in making our decisions about how much and when to pay participants: (1) the primary demand on participants in this study is time and we wanted to compensate participants adequately for this time; (2) the amount of money is not intended to be coercive; (3) we wanted to include participation from subjects across a full range of socioeconomic status. Participants were informed that the research is voluntary; that they were free to stop their participation at any time; participation did not impact their other services in any way; and that they would be paid for their participation. The consent form described the study procedures, including saliva collection for DNA, interviews about trauma history and symptoms, and the acoustic startle procedure. The potential risks and discomforts were also described and reviewed with participants before they signed the informed consent form. We anticipated that some women could experience distress after the FC task. As established in the research protocol of the Grady Trauma Project, all women were able to openly discuss any possible distress with the interviewers, encouraged to address it with their clinicians, and were given phone numbers for further contact. The interviewers were able to identify women in need of psychiatric treatment and made direct referrals to the Grady Healthcare System and other local treatment providers. Women with previously undiagnosed mental disorders, not resulting from their participation in the study, received medical treatment following the Grady Hospital mission regardless of insurance status or ability to pay. Patients were paid $60 in the FPS experiment. All participants provided written consent approved by the Emory University Institutional Review Board and Grady Research Oversight Committee.

#### Assessments in clinical populations

For Fig. 6, Supplementary Fig. 8, and Supplementary Tables 1–3, women received an initial assessment in the emergency department where clinical and demographic variables were collected. Post-traumatic symptoms were evaluated at around 3 weeks post trauma using the Acute Stress Disorder Interview (ASDI), which is a structured clinical interview with 19 items that assess dissociative, re-experiencing, avoidance, and hyperarousal symptoms^51^. The ASDI has good internal consistency (*r* = 0.90), and excellent sensitivity (91%) and specificity (93%) compared to independent clinical diagnosis^51^. Women received periodic clinical follow-ups for up to a year after sexual assault by a trained mental healthcare professional that monitored their disease course and detected the appearance of any mental disorder. In Supplementary Table 1, PTSD diagnoses were obtained in a retrospective analysis of clinical data, many women did not attend medical follow-ups before 1 year and therefore were accounted as unknown for PTSD diagnosis. Reasons to abandon the study included personal decision, migration, symptom attenuation or inability to reach them by phone.

For Fig. 7, Supplementary Fig. 9 and Supplementary Table 4, PTSD symptoms were assessed using the Modified PTSD Symptom Scale (PSS), which is a psychometrically valid self-report scale that assesses post-traumatic symptom severity during the last two weeks with 17 items^102^. The Childhood trauma questionnaire (CTQ) is a self-report tool used to assess three types of childhood abuse: sexual, physical, and emotional. For this study, the brief version of the CTQ was used with high reliability for this population^62^. Previous studies have shown its validity, internal consistency, and stability over time^103^.

#### Procedures in clinical populations

Menstrual cycle phase: For the allocation of women into menstrual cycle phases at the moment of trauma, we used the last menstrual period date, which was collected in the first visit to the ED. We used the mean age in our sample (30 ± 10.4 years) to calculate the duration of each menstrual cycle phase according to data from studies with large samples^104^. The segmentation into menstrual cycle phases was based on the hormonal profiles of interest (e.g., estradiol (E2) and progesterone (P4) levels). We discarded the cases with missing last menstrual period dates, irregular menstrual cycles, cycles lengths >35 days, or women using hormonal contraception. Women that were sexually abused between the initial or 9th day of the menstrual cycle (low levels of E2 and P4) were allocated into the early follicular phase; between the 10th and 17th day (high E2, low P4) in the late follicular phase; between the 18th and 27th day (high E2, high P4) in the mid-luteal phase and between 28th and 30th day (a decline of both, E2 and P4) in the late-luteal phase. For analyses, mid-luteal and late-luteal were collapsed into the luteal phase.

Grady Trauma Project: Research participants were approached in the waiting rooms of a public hospital’s primary care clinics while either waiting for their medical appointments or while waiting with others who were scheduled for medical appointments. After the subjects provided written informed consent, they participated in a verbal interview and donated saliva for genetic analyses^105^. DNA was extracted from saliva in Oragene collection vials (DNA Genotek Inc, Canada) using the DNAdvance kit (Beckman Coulter Genomics, USA). Genotyping was performed using the Omni-Quad 1 M Bead Chip. Quality control was performed by using the Psychiatric Genomics Consortium PTSD Workgroup guidelines^106^. Briefly, SNPweights software17 was used to assign ancestry. PLINK^107^ was used to perform quality control analyses such as SNPs that had a call rate <95% were removed; individuals with missingness with >2%, heterozygosity >0.2, and failing sex checks were removed; variants with significant deviation from Hardy–Weinberg proportions (*P* < 1 × 10^−6^ in controls and *P* < 1 × 10^−10^ in PTSD cases) were also excluded. Principal components for ancestry were calculated according to the PGC guidelines in each separate ancestry group^106^. We extracted the *ADCYAP1R*1 variant, rs2267735, from the genome-wide data. rs2267735’s HWE was *P* = 0.4 and *P* = 0.8 in EA and AA, respectively.

Fear-potentiated startle protocol: The protocol consisted of a fear acquisition and a FE phase. During fear acquisition, participants were initially presented to the CSs without any reinforcement to habituate them. Immediately after, they completed FC with the presentation of 36 trials divided into three blocks (12 trials per block) with four trials of each type (CS+, reinforced conditioned stimulus; CS-, non-reinforced conditioned stimulus; NA, a 40 ms, 108 dB noise probe alone). CSs consisted of colored shapes shown on a computer screen for 6 s. As in previous studies^59^, a 250 ms, 140 p.s.i. air blast directed to the larynx was used as the US. Intertrial intervals were randomized between 9 and 22 s. After the fear acquisition phase, participants were allowed to rest for 10 min before starting fear extinction phase. The Fear Extinction consisted of 72 trials divided into six blocks (12 trials per block) with four trials of each type (a non-reinforced CS+, CS−, and NA). None of the CS+ presentations were paired with an air blast US during the Fear Extinction phase.

Data acquisition and data analysis: Data were acquired with the electromyography (EMG) module of BIOPAC MP150 for Windows (Biopac Systems, Inc., USA). Data were then filtered, rectified, and smoothed using MindWare software suite (MindWare Technologies, Ltd., USA) and exported for statistical analyses. EMG signal was sampled at a frequency of 1 kHz and filtered with a low-frequency cutoff at 28 Hz and a high-frequency cutoff at 500 Hz. The measure of the acoustic startle response was captured as the maximum amplitude of the eyeblink muscle contraction appearing between 20 and 200 ms after the presentation of the startle probe. All amplitude values were used without coding low responses as zero. For data analyses, fear-potentiated startle was calculated using a Difference Score (startle magnitude to NA – startle magnitude to CS+/CS−) in each conditioning block. Fear Extinction was divided into early (first 4 CS+ trials) and late (last 4 CS+ trials). In an analysis of covariance (ANCOVA), fear extinction was analyzed as a repeated measures variable (two levels: early, late), and age and genotype as between-groups variables.

#### Data acquisition and analyses

Supplementary Data 1: Statistical analyses were performed with IBM SPSS 25.0. Repeated-measures ANOVA, one or two-way ANOVAs or Student’s *t* test (two-tailed) were used. Analysis of covariance (ANCOVA) was used for controlling for the effect of covariates over the dependent variable. Data were visualized, normality and equality of variance assumptions were calculated. For related sample analyses, CS trials in FC or mean FE per session were used as within-subject factors and treatment as between-subject factor. Greenhouse-Geisser correction was used when necessary. In all cases, if a statistically significant interaction was found, two-tailed pairwise comparisons were calculated. Bonferroni post hoc analyses were performed if necessary. Mann–Whitney’s *U* tests for independent samples or Kruskal Wallis’ H test were used for samples not meeting criteria for parametric analyses. Wald’s *χ*^2^ with pairwise comparisons was used in Generalized Linear Model for multifactorial analyses that did not meet normality or homoscedasticity. Additional individual comparisons were performed when appropriate. Pearson correlation coefficients were used for quantitative variables. The significance of association between categorical variables with <5 observations per group was calculated with the Fisher’s exact test. Data are presented as mean ± or +SEM and statistical significance was set at *P* < 0.05. Detection of outliers was performed using Grubb’s test and removed from analyses when necessary. A summary of the statistical analyses is shown in Extended Data. Procedural and schematics were created with BioRender.com. All graphs presented in the figures were designed using GraphPad Prism version 7.0 for Windows (GraphPad Software, USA).

### Reporting summary

Further information on research design is available in the Nature Research Reporting Summary linked to this article.

## Supplementary information

Supplementary Information

Description of Additional Supplementary Files

Supplementary Data 1

Reporting Summary

## Data Availability

No large-scale datasets were generated in this study. Source data are deposited in the Digital Repository of Documents from the Universitat Autónoma de Barcelona under accession code 10.5565/ddd.uab.cat/259560. Source data are provided with this paper.

## Source data

Source Data
