# Supplementary material for: PACAP-PAC1R modulates fear extinction via the ventromedial hypothalamus
Source: Nat Commun. 2022 Jul 28;13:4374. doi: 10.1038/s41467-022-31442-w (PMC9334354; doi:10.1038/s41467-022-31442-w)
Supplement: Supplementary file 1 — Supplementary Information [file 41467_2022_31442_MOESM1_ESM.pdf]

Supplementary Information for the article:

PACAP-PAC1R modulates fear extinction via the ventromedial hypothalamus. Velasco ER, Florido A, Flores Á, Senabre E, Gomez-Gomez A, Torres A, Roca A, Norrholm S, Newman EL, Das P, Ross RA, Lori A, Pozo OJ, Ressler KJ, Garcia-Esteve LL, Jovanovic T, Andero R.

Supplementary Figures

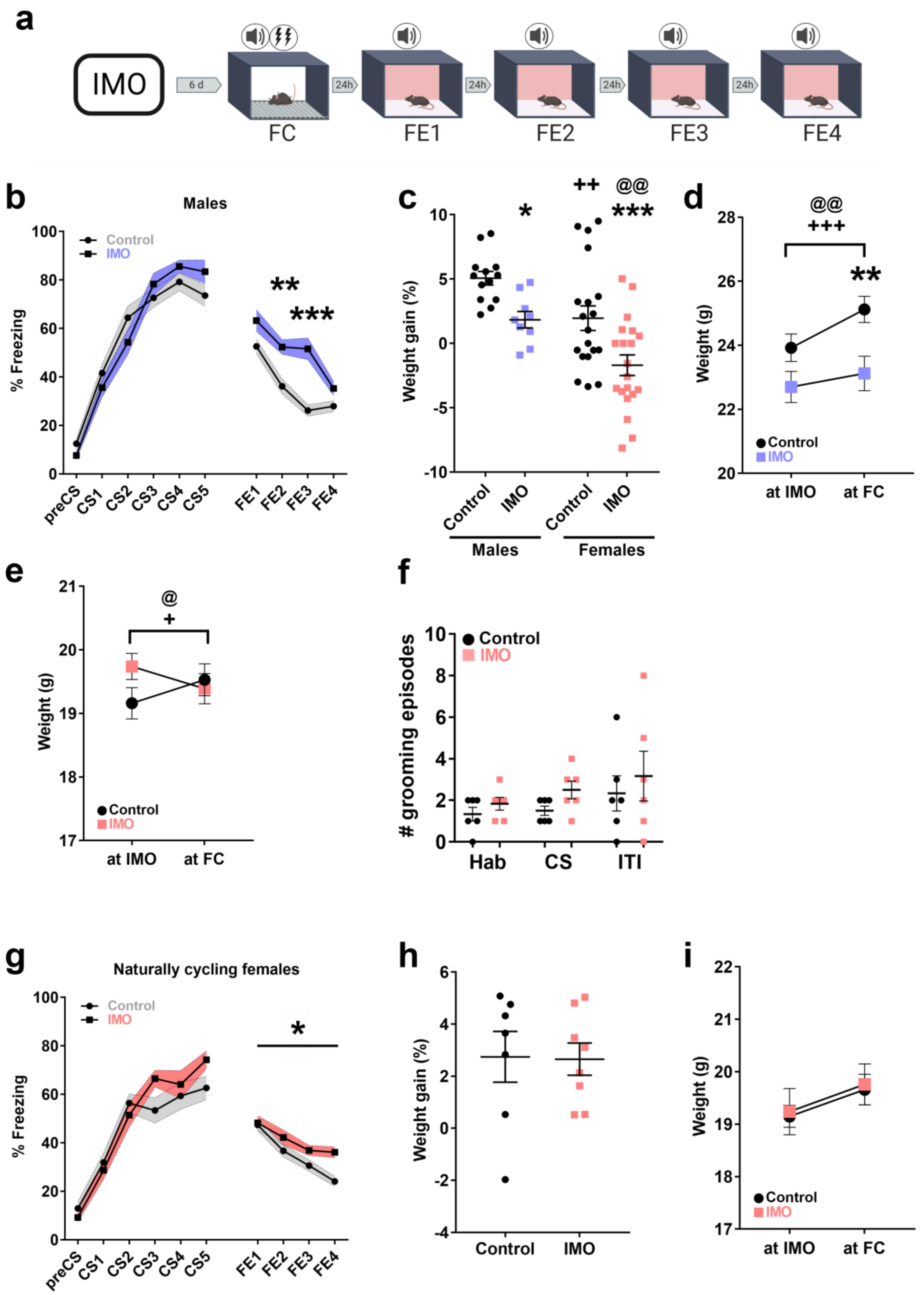

Supplementary Figure 1: IMO induces behavioral and physiological changes in males and female mice. a, Representation of the methods used to assess fear learning and fear extinction after IMO. **b,** Fear learning and fear extinction in males exposed to IMO vs compensatory handling (control: n=13, IMO=9) (FE session*stress: p=0.003). **c,** Comparison of the % weight gain of males and females (males control: n=13, IMO=9; females control: n=19, IMO: n=20) (sex*stress p<0.001). **d,** Impact of IMO on males' weight between IMO t1 and FC t2 (control: n=13, IMO=9) (time*stress p=0.001). **e,** Impact of IMO on females' weight between IMO t1 and FC t2 (control: n=19, IMO: n=20) (time*stress p=0.005). **f,** Impact of IMO on number of grooming episodes in females at habituation (Hab), tone presentations (CS), or inter-trial intervals (ITI) during FE1 session (n=6 per group). **g,** Fear learning and fear extinction in females with estrous cycle monitorization exposed to IMO vs compensatory handling, results are shown clustered by treatment (n=14 per group) (p=0.023). **h,** Impact of IMO on % weight gain in females with cycle monitorization shown clustered by treatment (control: n=7, IMO: n=8). **i,** Impact of IMO on cycle monitored females' weight between IMO t1 and FC t2 (control: n=7, IMO: n=8). Data are expressed as mean ± SEM. *p≤0.05, ** p≤0.01, *** p≤0.001. * IMO vs Control, @ IMO vs IMO, + Control vs Control. Asterisks above a line indicate significant main effect stress in repeated-measures ANOVA. In **b, d, e, g, i,** repeated measures ANOVA was used main effect stress, main effect FE session or CS or time, and FE session*stress or CS*stress or time*stress interactions were used. In **c,** a GzLM was conducted. In **f, h,** two-tailed t-tests were used. CS: conditioned stimulus, FC: fear conditioning, FE: fear extinction session, Hab: habituation, IMO: immobilization stress, ITI: intertrial interval.

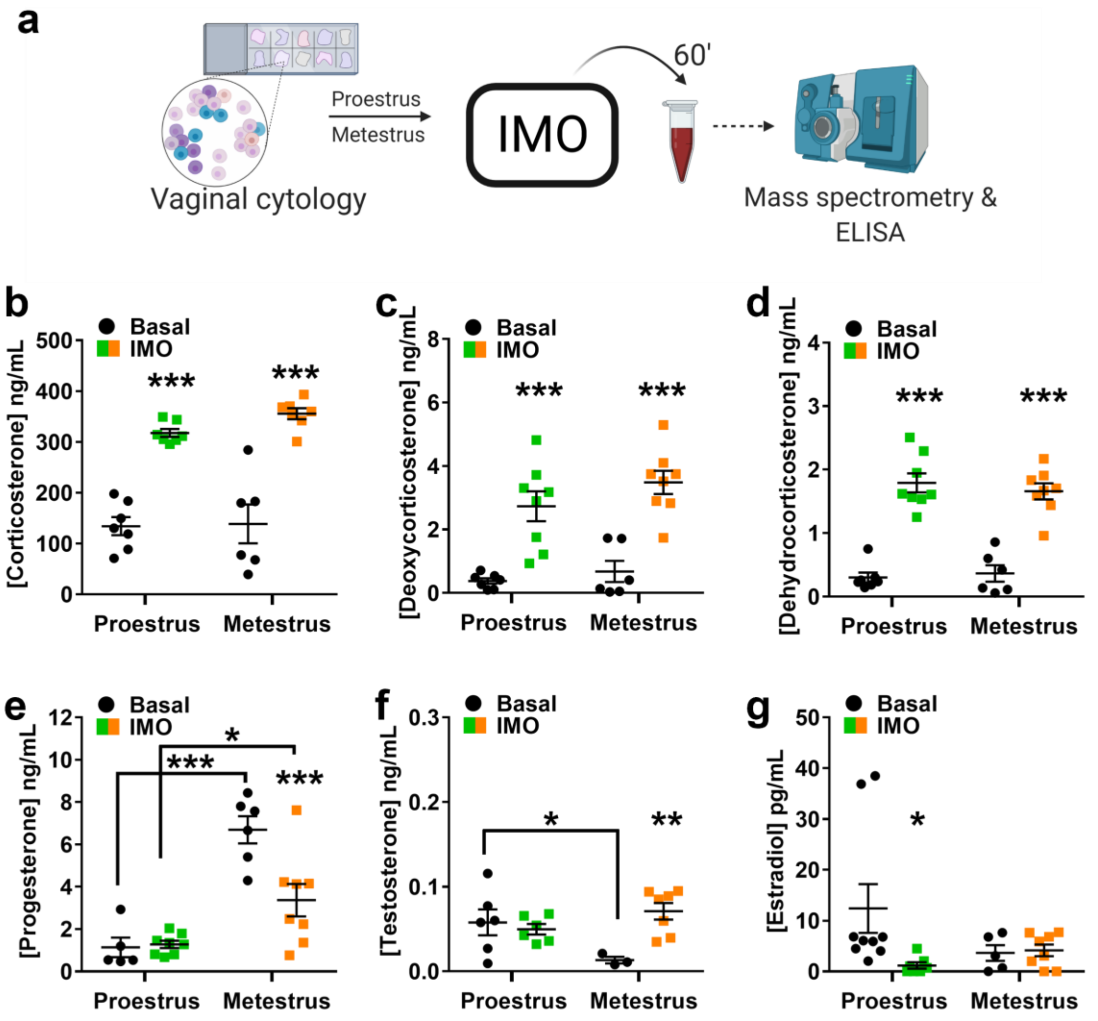

Supplementary Figure 2: Regulation of HPA and HPG hormones shortly after IMO in female mice during proestrus or metestrus. a, Methods used for cycle monitorization, stress procedure, and hormonal analyses. HPA hormonal regulation of **b,** Corticosterone (proestrus: n=7 per group; metestrus basal: n=6, IMO: n=7) (stress*estrus: p<0.001), **c,** Deoxycorticosterone (proestrus basal: n=7, IMO: n=8; metestrus basal: n=6, IMO: n=8) (stress*estrus: p<0.001), and **d,** Dehydrocorticosterone (proestrus basal: n=7, IMO: n=8; metestrus basal: n=6, IMO: n=8) (stress*estrus: p<0.001) 60 min after IMO in proestrus and metestrus females (n=5-8 per group). HPG hormonal regulation of **e,** Progesterone (proestrus basal: n=5, IMO: n=8; metestrus basal: n=6, IMO: n=8) (stress*estrus: p<0.001), **f,** Testosterone (proestrus n=6 per group; metestrus basal: n=3, IMO: n=7) (stress*estrus: p=0.004), and **g,** Estradiol (proestrus basal: n=9, IMO: n=7; metestrus basal: n=5, IMO: n=8) (stress*estrus: p=0.024), 60 min after IMO in proestrus and metestrus females. Data are expressed as mean ± SEM.*p≤0.05, ** p≤0.01, *** p≤0.001 asterisks above a line for specific comparisons. Data was analyzed with Generalized Linear Model (Wald's χ^2) with pairwise comparisons between groups and Bonferroni corrections. HPA: Hypothalamic-pituitary-adrenal axis, HPG: Hypothalamic-pituitary-gonadal axis, IMO: immobilization stress.

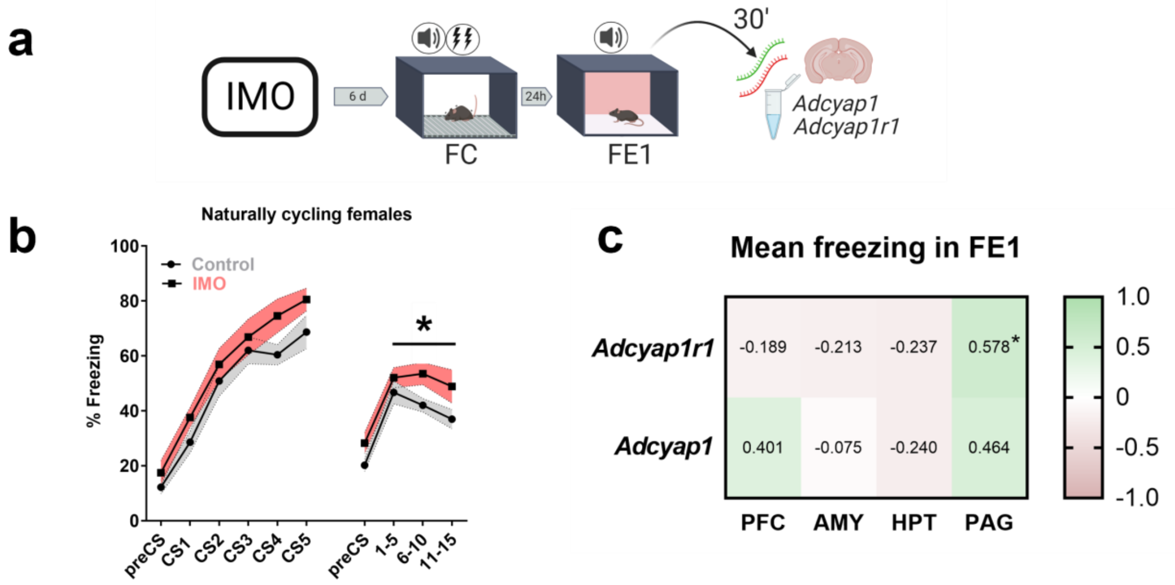

Supplementary Figure 3: Early FE in traumatized female mice and its association with *Adcyap1-Adcyap1r1* regulation. a, Representation of the methods used for the behavioral evaluation. **b,** Fear learning and early fear extinction in females exposed to IMO vs compensatory handling (control: n=12, IMO: n=9) (p=0.042). **c,** Correlation of mean freezing scores during FE1 with *Adcyap1* or *Adcyap1r1* mRNA levels. AMY- amygdala, HPT- hypothalamus, PAG- periaqueductal gray, PFC- prefrontal cortex. R values are shown, * signals significant results, magnitude of the correlation is depicted by a color heatmap. In **b,** main effect stress, main effect FE session or CS, and FE session*stress or CS*stress interactions were analyzed using repeated-measures ANOVA. Data are expressed as mean ± SEM. *$p \leq 0.05$. Asterisks above a line indicate main effect stress in repeated-measures ANOVA. In **c,** the Pearson correlation coefficient was used. CS: conditioned stimulus, FC: fear conditioning, FE: fear extinction, IMO: immobilization stress.

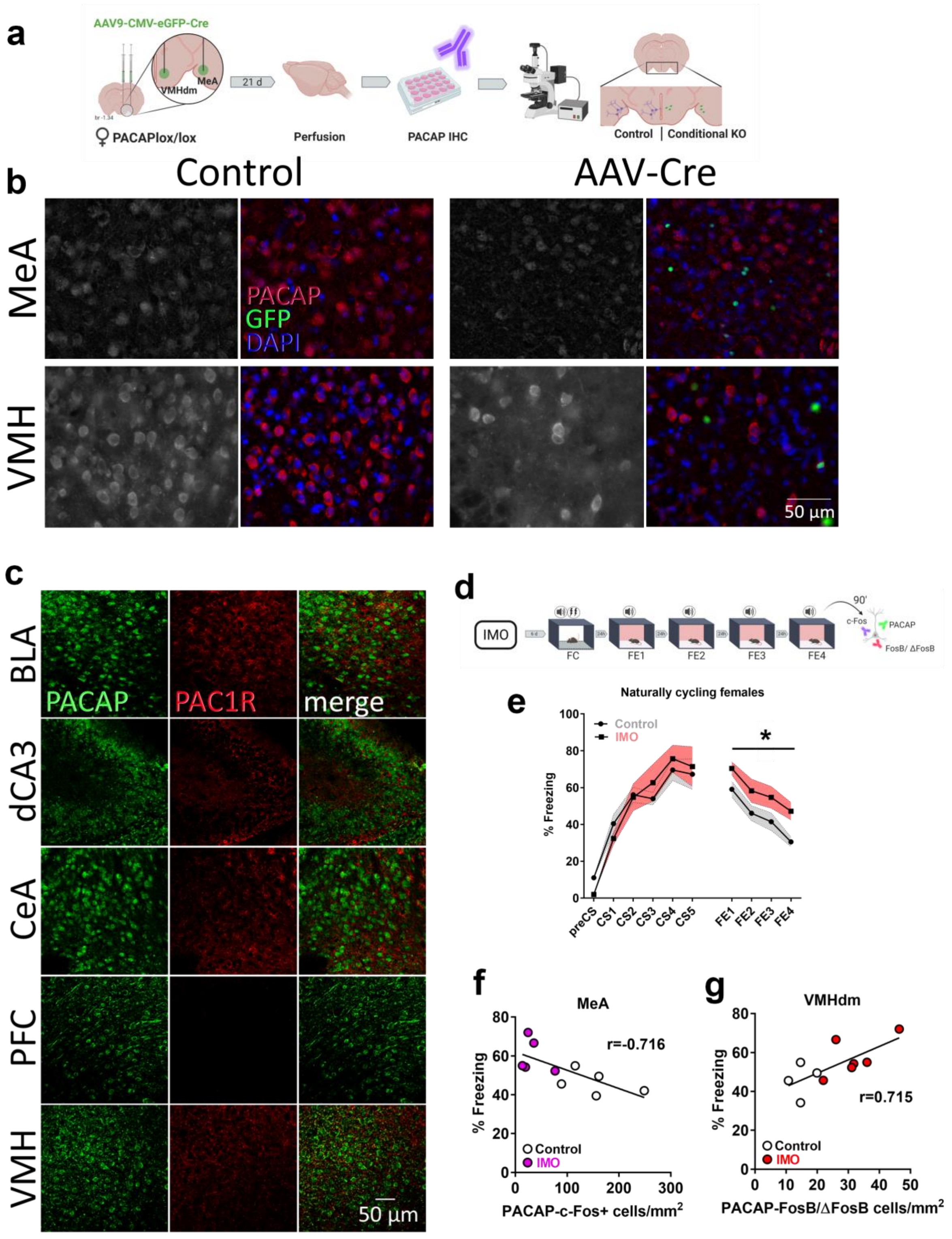

Supplementary Figure 4: PACAP antibody validation and relation of PACAP neuronal activity to freezing levels in mice. a, PACAP$^{lox/lox}$ mice were injected unilaterally with AAV9-CMV-eGFP cre into the MeA and VMHdm to conditionally delete *Adcyap1* on one side. IHC slices were prepared with T-4469 anti-PACAP antibody. The contralateral uninfected side served

as the control for PACAP expression. **b,** Intact PACAP immunolabels observed in MeA and VMHdm of non-injected side (Control) and fewer PACAP immunolabeled cells are observed in the MeA and VMHdm of the injected conditional KO side (AAV-Cre). **c,** Immunofluorescence study showing PACAP and PAC1R expression in areas of interest. BLA- basolateral amygdala, dCA3- dorsal CA3, CeA- central amygdala, PFC- prefrontal cortex, VMH- ventromedial hypothalamus. Scale bar 50µm. **d,** Schematic representation of the behavioral and immunohistochemical methods. **e,** Fear learning and fear extinction in females exposed to IMO vs compensatory handling (n=6 per group) (p=0.024). **f,** Correlation of % freezing in all FE sessions with PACAP-c-Fos+ cells/mm^2 in the MeA (n=5 per group). **g,** Correlation of % freezing in all FE sessions with PACAP-FosB/ΔFosB+ cells/mm^2 in the VMHdm (control: n=4, IMO: n=6). Data are expressed as mean ± SEM. *p≤0.05. In **e,** main effect stress, main effect FE session or CS, and FE session*stress or CS*stress interactions were analyzed using repeated-measures ANOVA. Asterisks above a line indicate significant main effect stress in repeated-measures ANOVA. In **f, g,** Pearson correlation coefficients were used. BLA: basolateral amygdala, CMV: cytomegalovirus, dCA3: dorsal CA3, CeA: central amygdala, GFP: green fluorescent protein, IHC: immunohistochemistry study, IMO: immobilization stress, MEA: medial amygdala, PFC: prefrontal cortex, VMH: ventromedial hypothalamus.

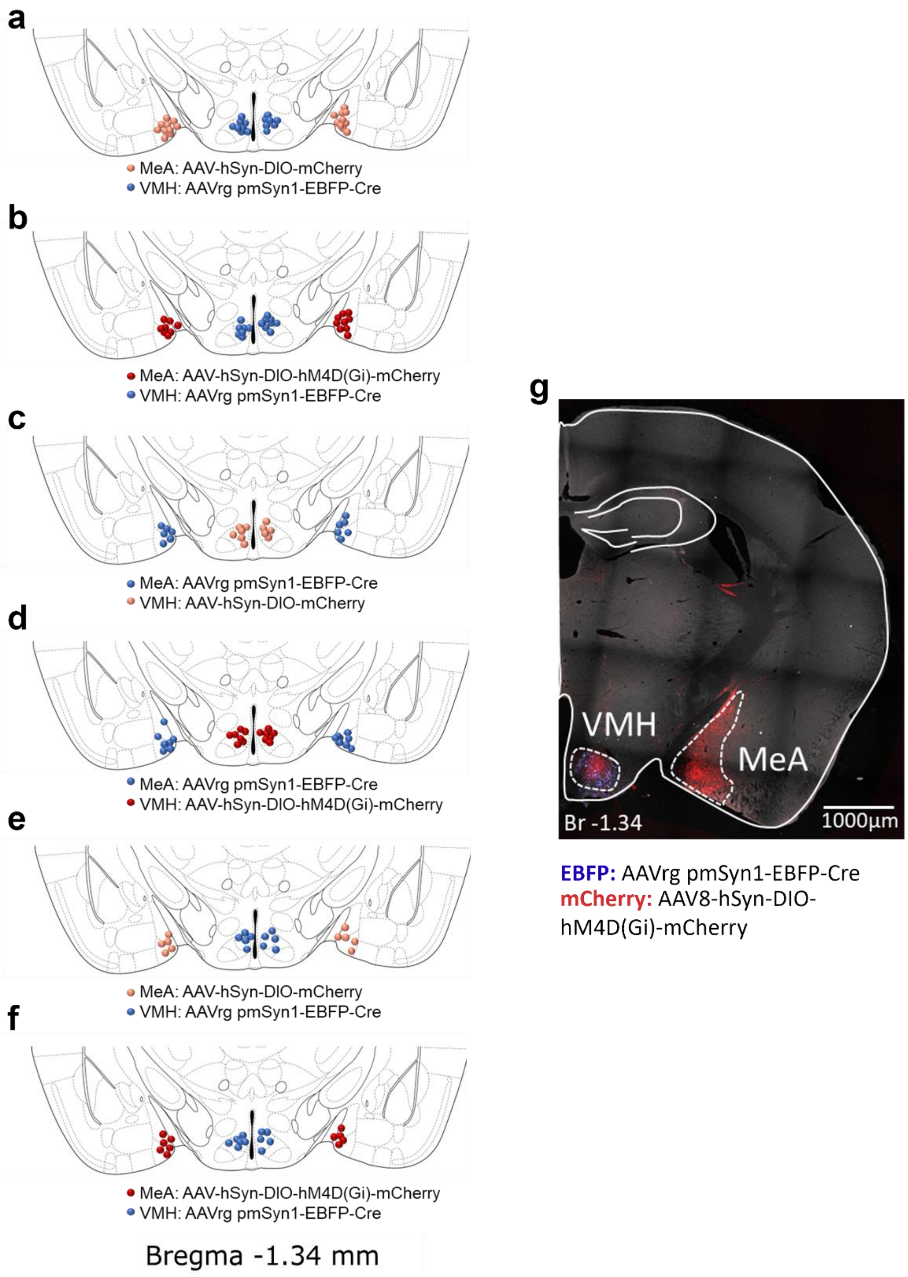

Supplementary Figure 5: Injection verification sites. a-f, Injections sites were verified for each animal under 20x objective magnification by direct visualization of needle trajectory and detection of EBFP or mCherry. Dots indicate the lowest point of the injector tip. Only animals with a prominent expression of both markers that were circumscribed to the area of interest and with at least an ipsilateral pair of accurate injections were included. Atlas images were adapted from Franklin & Paxinos (2007). **g,** Representative image of the viral vector reporter expression in the injection sites**.** EBFP or mCherry are visualized as blue or red respectively. Scale bar 1000 µm. AAV: adeno-associated virus, AAVrg: retrograde adeno-associated virus, Br: bregma, EBFP: Enhanced blue fluorescent protein, MeA: medial amygdala, VMH: ventromedial hypothalamus.

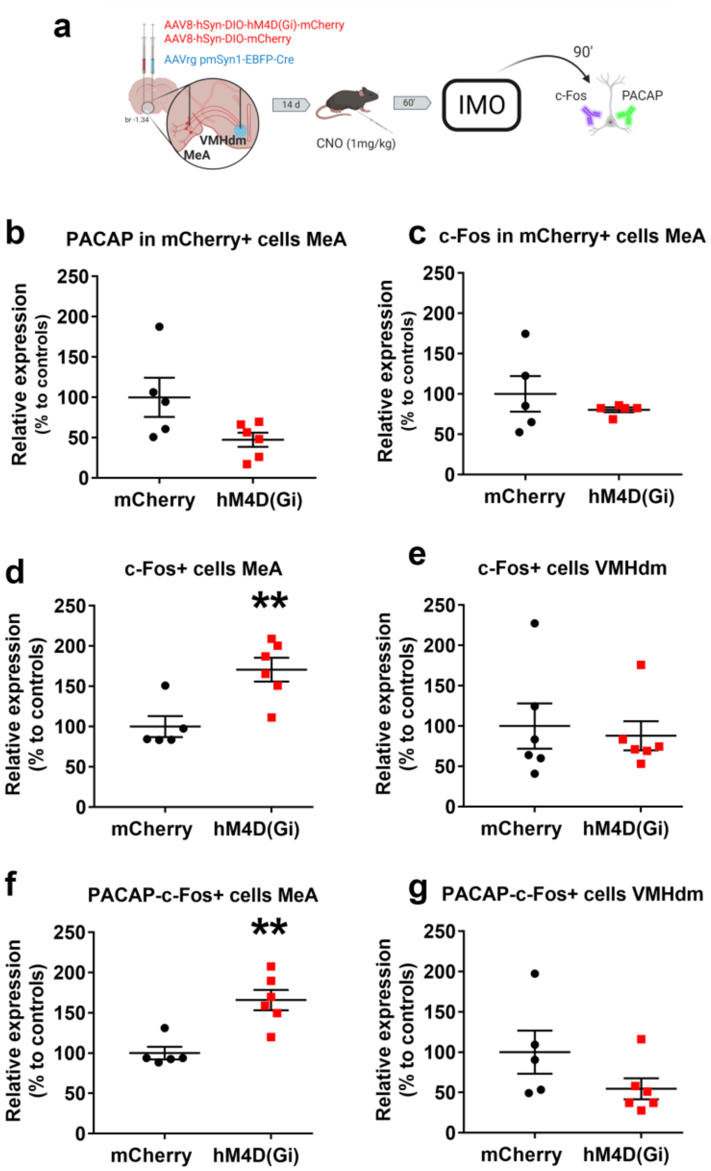

Supplementary Figure 6: Chemogenetic inhibition of the MeA to VMHdm circuit effects over PACAP levels and c-Fos expression shortly after IMO. a, Methods used to assess PACAP and c-Fos expression shortly after IMO (90 min) in animals with inhibited MeA to VMHdm circuitry (hM4D(Gi)) vs controls (mCherry). **b,** PACAP expression in mCherry+ cells in MeA (mCherry: n=5, hM4D(Gi): n=6). **c,** c-Fos-mCherry colocalization in MeA (n=5 per group). **d, e,** Chemogenetic inhibition effect over c-Fos expression in the MeA (mCherry: n=5, hM4D(Gi): n=6) and VMHdm (n=6 per group) (MeA: p=0.009). **f, g,** Chemogenetic inhibition effect over PACAP-c-Fos+ expression in MeA(mCherry: n=5, hM4D(Gi): n=6) and VMHdm (mCherry: n=5, hM4D(Gi): n=6) (MeA: p=0.009). Results are presented as relative expression to controls (mCherry) (n=5-6 per group). Data are expressed as mean ± SEM. **p≤0.01. Two-tailed t-tests or Mann Whitney U tests were used. CNO: clozapine N-oxide, MeA: medial amygdala, VMHdm: ventromedial hypothalamus dorsomedial nucleus.

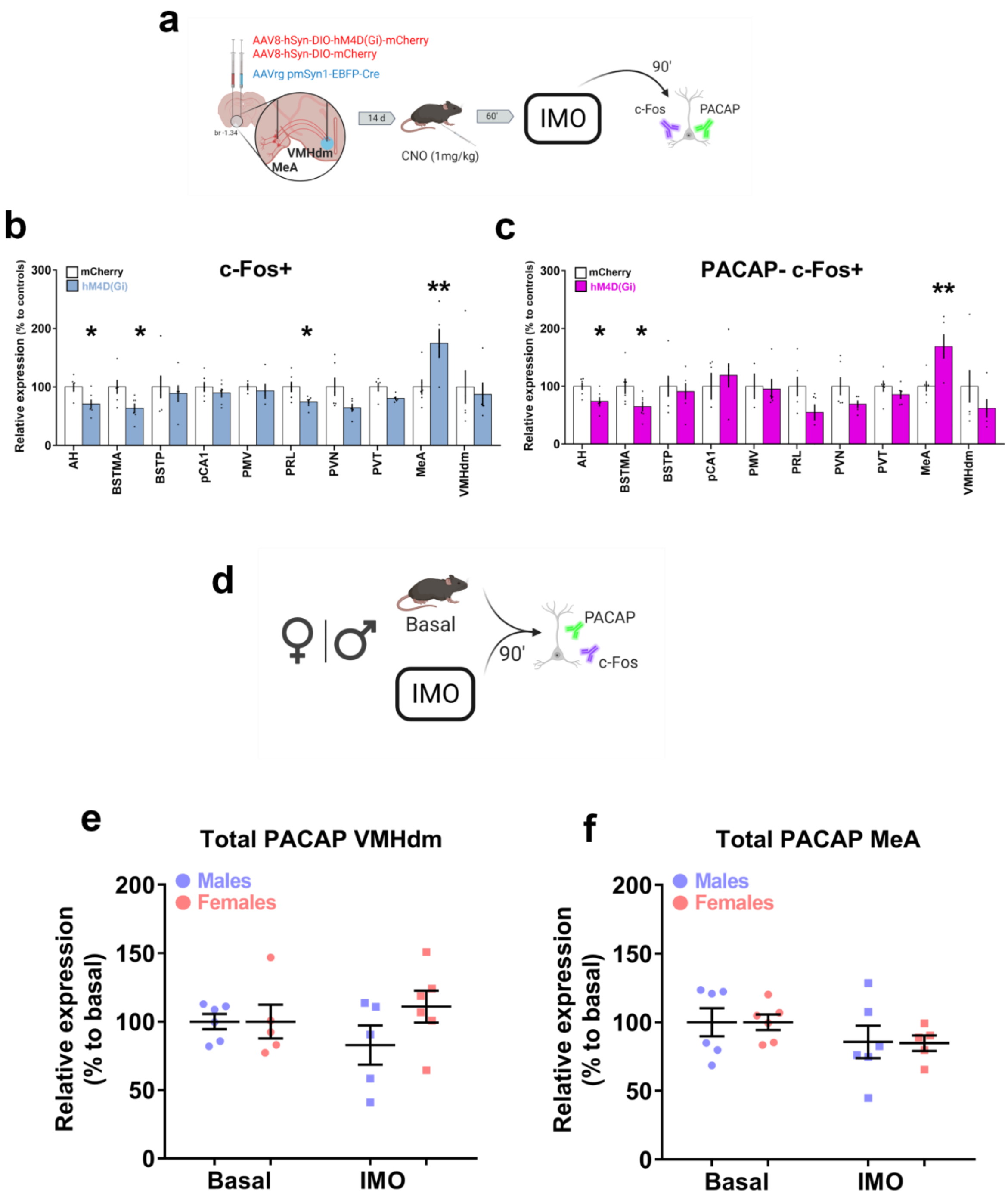

Supplementary Figure 7: Effect of the chemogenetic inhibition of MeA to VMHdm projections on other brain areas and sex differences in PACAP at basal levels and after IMO exposure. a, Methods used to assess PACAP and c-Fos expression shortly after IMO (90 min) in animals with inhibited MeA to VMHdm circuitry (hM4D(Gi)) vs controls (mCherry). **b,** Quantification of c-Fos+ neurons (AH, BSTMA, PRL, MeA n=6 per group (AH: p=0.029, BSTMA: p=0.030, PRL: p=0.019,MeA: p=0.003). **c,** Quantification of colocalization of PACAP-

c-Fos+ (AH, BSTMA, MeA n=6 per group) (AH: p=0.026, BSTMA: p=0.045, MeA: p=0.004). **d,** Methods used to assess PACAP levels in basal vs IMO males and females . **e,** Relative expression of PACAP between males and females in the VMHdm (males basal: n=6, IMO: n=5; females basal: n=5, IMO: n=6) and **f,** MeA (males basal: n=6, IMO: n=6; females basal: n=6, IMO: n=5). Results are presented as relative expression to controls, in **b, c** control is animals injected with mCherry; in **e, f,** controls are basal animals (n=5-6 per group). Data are expressed as mean ± SEM. *$p<0.05$, **$p\leq0.01$. For **b,** and **c,** two-tailed t-tests or Mann Whitney U tests were used. For **e,** and **f,** two-Way ANOVA was used. AH: Anterior Hypothalamus, BSTMA: Bed Nucleus of the Stria Terminalis Medial Anterior part, BSTP: Bed Nucleus of the Stria Terminalis Posterior part, CNO: clozapine N-oxide, IMO: immobilization stress, pCA1: Caudal CA1, PMV: Premamilary Nucleus Ventral part, PRL: Prelimbic Cortex, PVN: Paraventricular Nucleus of the Hypothalamus, PVT: Paraventricular Thalamus, MeA: Medial Amygdala, VMHdm: Ventromedial Hypothalamus dorsomedial nucleus.

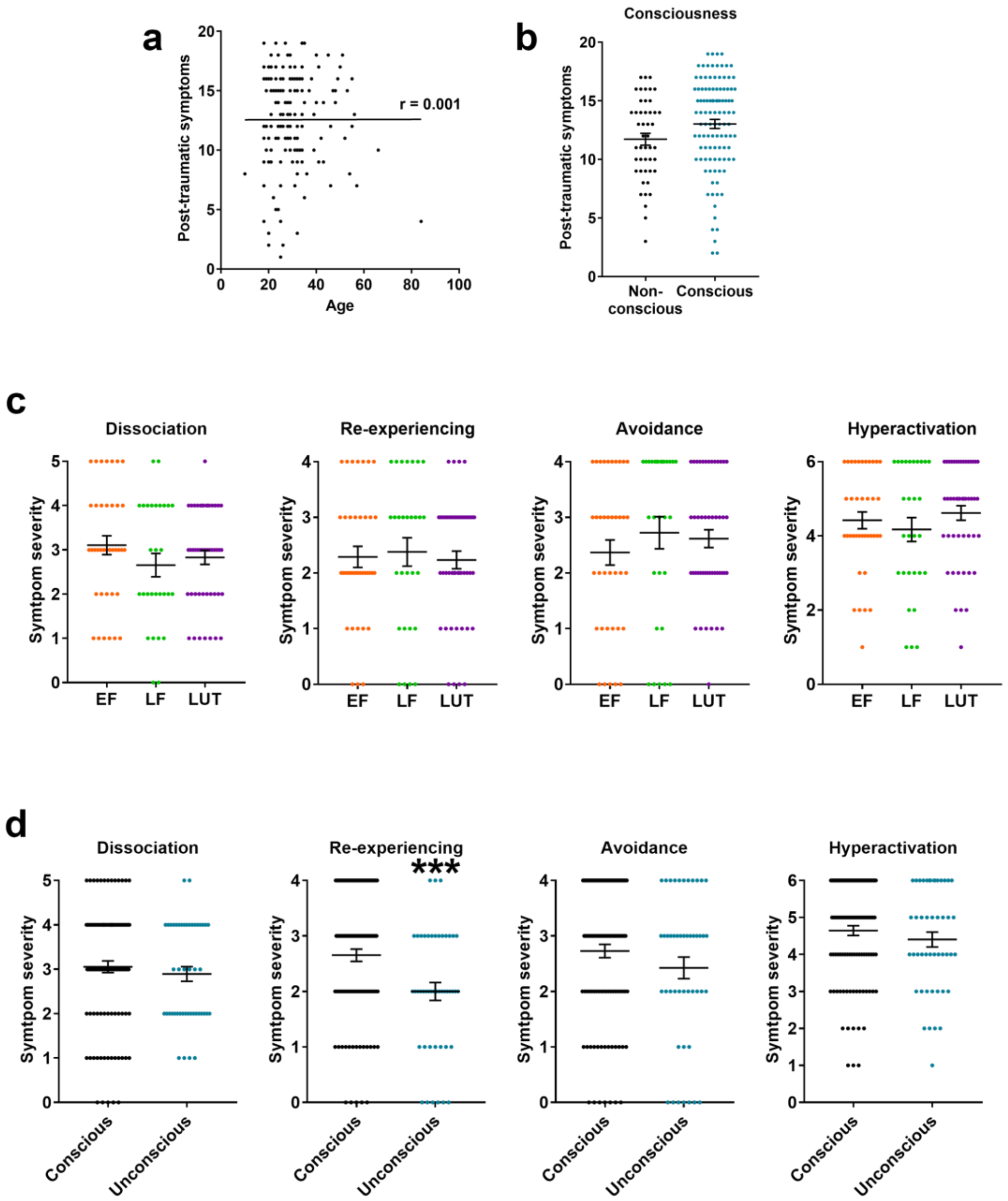

Supplementary Figure 8: Relation of posttraumatic symptom and sub-symptom severity with age, consciousness status and menstrual cycle phase. a, Correlation of age with posttraumatic symptom scores at 3 weeks post-trauma (n=170). **b,** Consciousness status and posttraumatic symptom severity at 3 weeks post-trauma (conscious: n=107, non-conscious: n=47). **c,** Sub-symptom scores (dissociation, re-experiencing, avoidance, hyperactivation) in women allocated in the distinct menstrual cycle phases at trauma (EF: n=38, LF: n=29, LUT: n=47). **d,** Sub-symptom scores in women that were conscious or non-conscious at trauma (conscious: n=107, unconscious: n=47) (Re-experiencing: p=0.001). Data are expressed as mean ± SEM. ***p≤0.001. In **a,** a Pearson correlation coefficient was used. In **b,** a two-tailed t-test was used. In **c,** Kruskal Wallis' H was used. In **d,** Mann-Whitney U Tests were used. EF: Early follicular, LF: Late follicular, LUT: luteal phase.

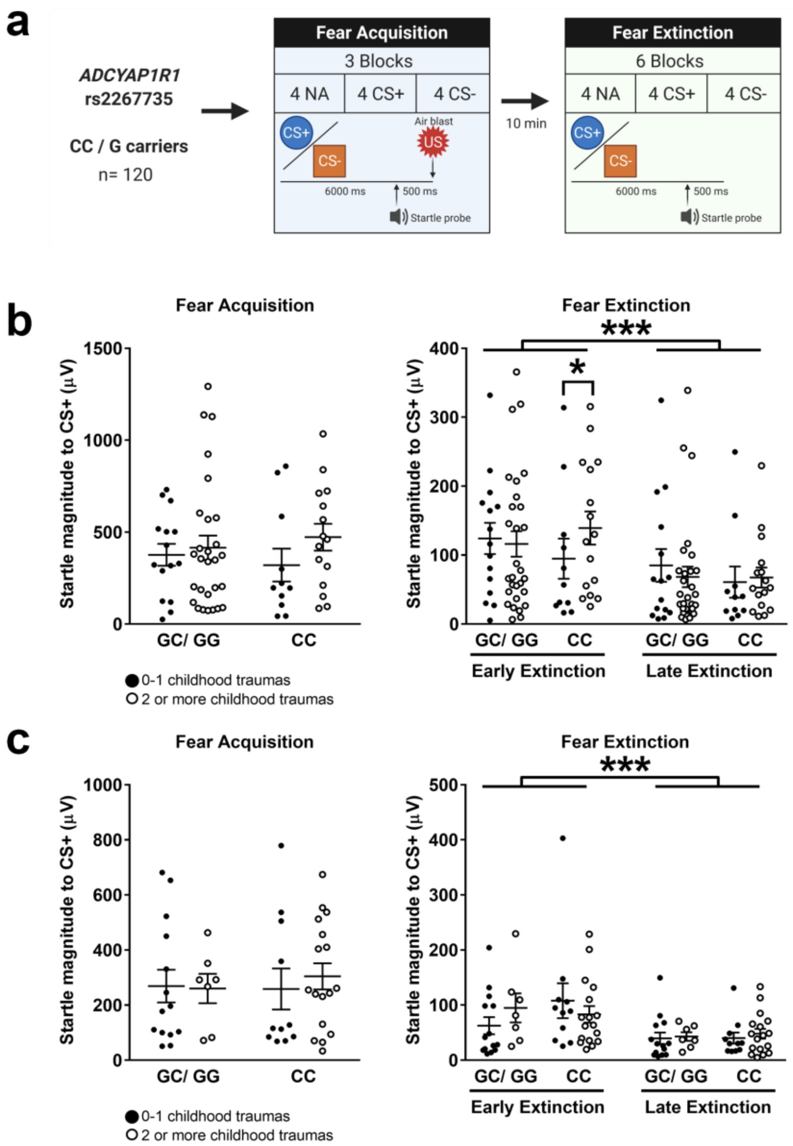

Supplementary Figure 9: *ADCYAP1R1* rs2267735 genotype during early FE in traumatized individuals. a, Methods used to assess of the relation of rs2267735 genotype with FE (n=71,49). **b, c,** Fear potentiated startle magnitude to CS+ in **b,** cycling women (≤40 years old) (n=71) (Early vs late extinction: $p<0.001$, genotype*childhood trauma: $p=0.030$) or **c,** women (>40 years old) (n=49) (Early vs late extinction: $p<0.001$) with CC or GC/GG genotypes during Fear Acquisition, Early FE and Late FE. Fear acquisition startle data are shown as the sum of the 3 acquisition blocks. Data are expressed as mean ± SEM. *$p<0.05$, ***$p\leq0.001$. Asterisks above a line indicate significant differences between Early and Late FE as assessed with Mann Whitney U tests. None of the CS+ presentations were paired with an US during the FE phase. CS+: reinforced conditioned stimulus, CS-: non-reinforced conditioned stimulus, NA: noise probe alone, US: unconditioned stimulus.

Supplementary Tables

Supplementary Table 1. Study participant demographics from Hospital Clínic cohort

Age (years), mean ± SD	30.2 ± 10.4
Pre-existing psychiatric disorders, n (%)	38 (22.4)
Anxiety or depressive disorders	25 (14.7)
Reproductive stage, n (%)	
Menopause	16 (9.4)
Reproductive years	154 (90.6)
Hormonal contraceptive use, n (%)	25 (16.4)
Menstrual cycle, n (%)	
Irregular cycles	13 (10.2)
Regular cycles (phases)	114 (89.7)
Early follicular	38 (33.3)
Late follicular	29 (25.4)
Luteal	47 (41.3)
**Trauma- related**	
Consciousness status, n (%)	
Conscious	107 (62.9)
Non-conscious	47 (27.6)
Unknown	16 (9.5)
Meets ASD diagnosis at 3 weeks, n (%)	
Yes	107 (62.9)
No	63 (37.0)
PTSD diagnosis 1-year follow-up, n (%)	
PTSD	16 (9.4)
No PTSD	72 (42.3)
Unknown	69 (40.6)
Comorbidities diagnosis 1-year follow-up, n (%)	
Anxiety disorder	12 (7.1)
Depressive disorder	19 (11.2)
Other	2 (1.2)
No comorbidities	70 (41.1)
Unknow	67 (39.4)
ASDI total score, mean ± SD	12.64 ± 4.09
Dissociation symptoms (0-5)	2.98 ± 1.31
Re-experiencing symptoms (0-4)	2.41 ± 1.16
Avoidance symptoms (0-4)	2.64 ± 1.27
Hyperarousal symptoms (0-6)	4.55 ± 1.42

Data are presented as mean ± SD or number of subjects and percentage, n (%). ASD: acute stress disorder, ASDI: Acute Stress Disorder Interview, PTSD: posttraumatic stress disorder.

Supplementary Table 2. Proportion of women with history of trauma exposure from Hospital Clínic cohort

Type	Yes, n (%)	No, n (%)	Missing data, n (%)
CSA – Childhood sexual abuse	56 (32.9)	81 (47.6)	33 (19.4)
CEA – Childhood emotional abuse	44 (25.8)	91 (53.5)	35 (20.5)
CPA – Childhood physical abuse	55 (32.3)	82 (48.2)	33 (19.4)
PSAA – Previous SA in adulthood	30 (17.6)	113 (66.4)	27 (15.8)
PAA – Previous aggression in adulthood (non-SA)	35 (20.5)	128 (75.2)	7 (4.11)

Data are presented as number of subjects and percentage, n (%). SA: sexual abuse.

Supplementary Table 3. Follow-up (in days) of sexually abused women from Hospital Clínic cohort

Follow-up (days)	Mean ± SD	Median (IQR)
First contact	10.5 ± 7.6	9 (12)
First follow-up after ASDI	45.2 ± 11.1	43 (16)
Days after aggression to PTSD diagnosis	168.2 ± 217.3	75 (174)
Last follow-up in all women	221.7 ± 289.4	98 (237)
Last follow-up in women with PTSD	548.0 ± 415.0	491 (749)

Data are presented as mean ± SD or median (range). ASDI: Acute Stress Disorder Interview, IQR- interquartile range, PTSD: posttraumatic stress disorder.

Supplementary Table 4. Study participant demographics from Grady Trauma Project cohort

	**Mean ± SD**
Age, mean (range)	38.59 (18-62)
Race, n (%)	
African American	117 (97.5)
Other	3 (2.5)
CTQ, mean ± SD	46.11 ± 19.83
TEI	15.49 ± 12.82
PSS Total	15.49 ± 12.82
PSS Re-experiencing, mean ± SD	3.72 ± 3.65
PSS Avoidance	6.41 ± 6.04
PSS Hyperarousal	5.38 ± 4.50
Genotype G carrier, n (%)	65 (54.2)

Data are presented as mean ± SD or number of subjects and percentage (n (%)). CTQ: childhood trauma questionnaire, PSS: PTSD symptom scale, TEI: traumatic events inventory.
