# Supplementary material for: PACAP-PAC1R modulates fear extinction via the ventromedial hypothalamus
Source: Nat Commun. 2022 Jul 28;13:4374. doi: 10.1038/s41467-022-31442-w (PMC9334354; doi:10.1038/s41467-022-31442-w)
Supplement: Supplementary file 2 — Description of Additional Supplementary Files [file 41467_2022_31442_MOESM2_ESM.pdf]

Description of Additional Supplementary Files

Supplementary Data 1: Statistics performed for PACAP-PAC1R modulates fear extinction via the ventromedial hypothalamus.
