# Supplementary material for: PACAP-PAC1R modulates fear extinction via the ventromedial hypothalamus
Source: Nat Commun. 2022 Jul 28;13:4374. doi: 10.1038/s41467-022-31442-w (PMC9334354; doi:10.1038/s41467-022-31442-w)
Supplement: Supplementary file 4 — Reporting Summary [file 41467_2022_31442_MOESM4_ESM.pdf]

nature research

Corresponding author(s): Raul Andero Gali
Last updated by author(s): May 27, 2022

Reporting Summary

Nature Research wishes to improve the reproducibility of the work that we publish. This form provides structure for consistency and transparency in reporting. For further information on Nature Research policies, see our Editorial Policies and the Editorial Policy Checklist.

Statistics

For all statistical analyses, confirm that the following items are present in the figure legend, table legend, main text, or Methods section.

n/a	Confirmed	
☐	☒	The exact sample size (*n*) for each experimental group/condition, given as a discrete number and unit of measurement
☐	☒	A statement on whether measurements were taken from distinct samples or whether the same sample was measured repeatedly
☐	☒	The statistical test(s) used AND whether they are one- or two-sided<br>*Only common tests should be described solely by name; describe more complex techniques in the Methods section.*
☐	☒	A description of all covariates tested
☐	☒	A description of any assumptions or corrections, such as tests of normality and adjustment for multiple comparisons
☐	☒	A full description of the statistical parameters including central tendency (e.g. means) or other basic estimates (e.g. regression coefficient) AND variation (e.g. standard deviation) or associated estimates of uncertainty (e.g. confidence intervals)
☐	☒	For null hypothesis testing, the test statistic (e.g. *F*, *t*, *r*) with confidence intervals, effect sizes, degrees of freedom and *P* value noted<br>*Give P values as exact values whenever suitable.*
☒	☐	For Bayesian analysis, information on the choice of priors and Markov chain Monte Carlo settings
☒	☐	For hierarchical and complex designs, identification of the appropriate level for tests and full reporting of outcomes
☐	☒	Estimates of effect sizes (e.g. Cohen's *d*, Pearson's *r*), indicating how they were calculated

Our web collection on statistics for biologists contains articles on many of the points above.

Software and code

Policy information about availability of computer code

Data collection	Freezing v1.3.04 software (Panlab-Harvard, Barcelona, Spain) was used for controlled delivery of tones and shocks and registration of freezing behaviour through a high sensitivity weight transducer system. TargetLynx module of the MassLynx software v4.1 software (Water Associates, USA) was used for quantification by external calibration approach of plasma. Fiji for Windows v1.53c was used for confocal image processing. LungJ Plugin plugin version 0.5.1 was used to create and apply mask, identification and quantification of colocalization . 7500FAST v2.0.6 (Thermo Fisher, Barcelona, Spain) software was employed as controller for thermocycler 700FAST for quantification ADCYAP1R1/ ADCYAP1 mRNA. Zeiss LSM700 Confocal microscope was controlled with ZEN v2010 software. Leica TCS SP5 Confocal microscope was controlled by LAS X v2.7.3.9727 software. BIOPAC MP150 for Windows (Biopac Systems, Inc., Aero Camino, CA) was used for the acquisition and extraction of FPS data. Data were then filtered, rectified, and smoothed using MindWare software suite (MindWare Technologies, Ltd., Gahanna, OH) and exported for analyses. SNPweights software was used to assign ancestry and PLINK was used to perform quality control analyses.
Data analysis	IBM SPSS Statistics v25.0 (IBM, Madrid, Spain) was used for data analyses. Procedural and schematics were created with BioRender.com. Representative images were created with Microsoft® PowerPoint® for Microsoft 365 MSO (16.0.14326.20936) 64-bit. All graphs presented in the figures were designed using GraphPad Prism version 7.0 for Windows (GraphPad Software, USA)

For manuscripts utilizing custom algorithms or software that are central to the research but not yet described in published literature, software must be made available to editors and reviewers. We strongly encourage code deposition in a community repository (e.g. GitHub). See the Nature Research guidelines for submitting code & software for further information.

Data

Policy information about availability of data

All manuscripts must include a data availability statement. This statement should provide the following information, where applicable:

- Accession codes, unique identifiers, or web links for publicly available datasets
- A list of figures that have associated raw data
- A description of any restrictions on data availability

No large-scale datasets were generated in this study. Source data are deposited in the Digital Repository of Documents from the Universitat Autónoma de Barcelona under accession code https://doi.org/10.5565/ddd.uab.cat/259560

Field-specific reporting

Please select the one below that is the best fit for your research. If you are not sure, read the appropriate sections before making your selection.

☒ Life sciences ☐ Behavioural & social sciences ☐ Ecological, evolutionary & environmental sciences

For a reference copy of the document with all sections, see nature.com/documents/nr-reporting-summary-flat.pdf

Life sciences study design

All studies must disclose on these points even when the disclosure is negative.

Sample size	All sample sizes were utilized based on previous research using similar techniques in absence of power analysis (PMID: 24976214, PMID: 26568307, PMID: 11487652, PMID: 10938577, PMID: 32034128, PMID: 31661603, PMID: 30705647, PMID: 24484701, PMID: 30439419, PMID: 25451788).
Data exclusions	Exclusion criteria were pre-established based on our ethics protocols approved for experiments in mice ref. CEEAH 3603 and biosecurity protocols 345-16 and 407-17. Animals that were an statistically significant outlier were excluded from the dataset according to Grubb's test. Animals without an ipsilateral pair of injections (retro-Cre/ Dreadds) were discarded. Also, we discarded animals with little viral infections (<20 neurons mCherry+). According to our ethics protocol, animals that presented disconfort during the different procedures were discarded from the experiments and all animals used in the reported study presented an optimal health state. For menstrual cycle human cohort, we discarded women using oral contraceptives, reporting irregular menstrual cycles or in menopause/perimenopause.
Replication	No replications were carried out.
Randomization	Mice were randomly allocated to experimental groups following an alternating order based on housing boxes. e.g. box1, 3, 5, 7 to experimental condition 1. box2, 4, 6, 8 to experimental condition 2. The different experimental conditions were alternated during experiments to avoid any effect of time or day during experiments. Mice from different experimental conditions in the same experiments were run at the same time in identical fear chambers, and chambers for experimental conditions were counterbalanced to avoid any bias in the results. Women in the Grady Trauma Project were recruited randomly from the waiting room in the general medical clinics of Grady Hospital in Atlanta, GA as part of a broader Fear Extinction study. Experimental groups were unkown for researchers and participants since SNP genotyping experiments were conduced once the FE task was completed. Covariates were controlled with an ANCOVA for the analyses. In the Hospital Clinic cohort, demographic, menstrual cycle and trauma-related data were collected at the ED. Follow-up visits were carried out for up to a year and menstrual cycle effects over posttraumatic symptom intensity were analyzed retrospectively once the follow-up visits had finished. Variables related to trauma or others known to influence posttraumatic symptom intensity were tested independently by introducing them as additional between-subject factors.
Blinding	Animals were given a code before the experiments and this code was not accesible to the experimenter until data analyses. Further, freezing data were collected using automated software with no intervention of experimenters. For biochemical studies, the experimenters that performed biochemical determinations had no access to the different groups or treatments and they were different from the one that administered the different treatments. Human analyses were peformed blind to group.

Reporting for specific materials, systems and methods

We require information from authors about some types of materials, experimental systems and methods used in many studies. Here, indicate whether each material, system or method listed is relevant to your study. If you are not sure if a list item applies to your research, read the appropriate section before selecting a response.

Materials & experimental systems

n/a	Involved in the study	
[ ]	[x]	Antibodies
[x]	[ ]	Eukaryotic cell lines
[x]	[ ]	Palaeontology and archaeology
[ ]	[x]	Animals and other organisms
[ ]	[x]	Human research participants
[x]	[ ]	Clinical data
[x]	[ ]	Dual use research of concern

Methods

n/a	Involved in the study	
[x]	[ ]	ChIP-seq
[x]	[ ]	Flow cytometry
[x]	[ ]	MRI-based neuroimaging

Antibodies

Antibodies used

Sheep anti-c-Fos (1:500, Abcam, Cambridge, UK, ab6167)
Mouse anti-FosB/ delta FosB [IgG1-83B1138] (1:2000, Abcam, Cambridge, UK, ab11959)
Rabbit anti-PACAP-38 (1:1000, Peninsula Laboratories, T4469)
Mouse anti-vGLUT2 [IgG1-8G9.2] (1:300, Abcam, Cambridge, UK, ab79157)
Mouse anti-PAC1R [IgG1-kappa] (1:100, Abcam, Cambridge, UK, ab54980)
Alexa Fluor® 488 AffiniPure F(ab')$_2$ Fragment Donkey Anti-Rabbit IgG (H+L) (1:1000, Jackson Immunoresearch, Madrid, Spain, 715-545-150).
Rhodamine Red™-X (RRX) AffiniPure Donkey Anti-Mouse IgG (H+L) (1:1000, Jackson Immunoresearch, Madrid, Spain, 715-295-150)
Alexa Fluor® 647 AffiniPure F(ab')$_2$ Fragment Donkey Anti-Mouse IgG (H+L) (1:1000, Jackson Immunoresearch, Madrid, Spain, 715-606-150)

Validation

Antibodies are well-characterized and commercially available with specificity tested by manufacturers. We provide the links below to the validation profiles for each antibody in conjunction with citations from other works using them. Furthermore, we performed usual validation of the antibodies for fluorescence microscopy with positive and negative controls, along with previous images obtained in our lab or published elsewhere. Adequate dilutions were obtained following manufacturer's instructions and also after titration tests for dilution to obtain the desired signal. Please, find below references (PMID#) and validation profiles for the antibodies used:

Sheep anti-c-Fos. Specificity: Extensive immunohistochemical analysis shows that it has high specificity to cFOS. 4 references available. Details at: (https://www.abcam.com/c-fos-antibody-ab6167.html; PMID: 32043970).

Mouse anti-FosB/ delta FosB. Validation in IF antibody immunostaining in mouse brain cortex showing the striatum. Tissue was fixed with paraformaldehyde, before incubation with the antibody (1/3000). 29 refs available. Details at: (https://www.abcam.com/fos-b-antibody-83b1138-ab11959.html; PMID: 32901027)

Mouse monoclonal anti-vGLUT2. Validation in IF at 1/300 dilution, staining VGLUT2 by immunohistochemistry using ABC with NiDAB on floating mouse brain tissue sections. 37 refs available. Details at: (https://www.abcam.com/vglut2-antibody-8g92-ab79157.html; PMID: 30021416)

Mouse anti-PAC1R [IgG1-kappa]. Validated ICC/IF stained SKNSH cells at 5µg/ml. 2 references available. Details at: (https://www.abcam.com/pacap-receptor-antibody-ab54980.html)

Rabbit anti-PACAP-38 (http://www.bma.ch/en/products/t-4469; validation performed in our paper with a conditional KO using PACAP flox/flox mice and AAV9-CMV-eGFP-Cre (Addgene 105545-AAV9) See Extended Data Fig. 4a).

Alexa Fluor® 488 AffiniPure F(ab')$_2$ Fragment Donkey Anti-Rabbit IgG (H+L) (https://www.jacksonimmuno.com/catalog/products/715-545-150; PMID: 33504795)
Rhodamine Red™-X (RRX) AffiniPure Donkey Anti-Mouse IgG (H+L) (https://www.jacksonimmuno.com/catalog/products/715-295-150; PMID: 31314751)
Alexa Fluor® 647 AffiniPure F(ab')$_2$ Fragment Donkey Anti-Mouse IgG (H+L) (https://www.jacksonimmuno.com/catalog/products/715-606-150; PMID: 31736686)

Animals and other organisms

Policy information about studies involving animals; ARRIVE guidelines recommended for reporting animal research

Laboratory animals

Male and naturally cycling female wild-type C57BL/6J purchased from Charles River (Spain) were regularly used for all experiments (8 weeks old). Antibody validation experiments were carried out on 6-month-old PACAPflox/flox females.

Wild animals

Not employed

Field-collected samples

Not collected

Ethics oversight

Ethics protocols were approved for the experiments in mice ref. CEEAH 3603 and biosecurity protocols 345-16 and 407-17. All

procedures were approved by the Committee of Ethics of the Universitat Autònoma de Barcelona and the Generalitat de Catalunya. They were also carried out in accordance with the European Communities Council Directive (2010-63-UE) and Spanish legislation (RD 53/2013). IACUC protocol for the mice when they were alive was McLean-based, and 2017N000228.

Note that full information on the approval of the study protocol must also be provided in the manuscript.

Human research participants

Policy information about studies involving human research participants

Population characteristics	For women from the Grady Trauma Project, inclusion criteria were willingness to participate and the ability to understand the informed consent form. Exclusion criteria were active psychosis and major medical illnesses as assessed by history and physical examinations conducted by licensed physicians. Participants with a positive toxicology urine test for cocaine were also excluded. All participants were screened for hearing impairments with an audiometer (Grason-Stadler, Model GS1710), and the ones not able to detect tones of 30 dB(A)SPL at frequencies ranging from 250–4000 Hz were not included in the study. For women in the Hospital Clínic de Barcelona, inclusion criteria were willingness to participate, being older than 18 years, and being a victim of a recent sexual assault. Exclusion criteria were language barriers, being a tourist, having mental disabilities or active psychosis, and women abandoning the study before posttraumatic symptom assessment at the 3rd-week post-trauma. For the final cohort, we additionally excluded women with missing last menstrual period dates, women in menopause, and women with irregular menstrual cycles for the final cohort of participants. Patients were paid $60 in the FPS experiment. Participants of the Hospital Clinic de Barcelona cohort did not receive payment as the study was part of their treatment plan. In both cohorts written informed constents were obtained before study enrollment.
Recruitment	Women in the Grady Trauma Project were recruited randomly from the waiting room in the general medical clinics of Grady Hospital in Atlanta, GA as part of a broader Fear Extinction study. Women from the Hospital Clínic de Barcelona were recruited from the emergency department as part of a specialized protocol that provides first-aid care in sexually abused women. We must acknowledge that our samples may be biased towards women who actively seek medical care for trauma, leaving a proportion of women not seeking medical care underrepresented in our study.
Ethics oversight	Emory University Institutional Review Board and Grady Research Oversight Committee and the Ethics Committee of Clinical Research in the Hospital Clinic de Barcelona

Note that full information on the approval of the study protocol must also be provided in the manuscript.
